# Burden and temporal trends of non-communicable diseases from 1990 to 2021 and prediction to 2035 in the group of twenty countries: a systematic analysis of the Global Burden of Disease Study 2021

**DOI:** 10.3389/fmicb.2026.1851514

**Published:** 2026-05-19

**Authors:** Li Song, Shuotong Li, Jingjing Wang, Xiangwen Li, Rongqiang Zhang, Qiling Liu, Yuxuan Liu, Zhili Chang

**Affiliations:** 1 School of Public Health, Shaanxi University of Chinese Medicine, Shaanxi, Xianyang, China; 2 College of Economics, Shenzhen University, Guangdong, Shenzhen, China; 3 Nanjing Hankai Academy, Nanjing, China; 4 Department of Medical, Nanjing Geneseeq Technology Inc., Nanjing, China

**Keywords:** Global Burden of Disease, group of twenty, non-communicable diseases, prediction, temporal trends

## Abstract

**Background:**

Non-communicable diseases (NCDs) represent a major global public health threat, closely linked to disruptions in host–microbe symbiosis and metabolic homeostasis. The Group of Twenty (G20) comprises the world’s major economic powers with diverse developmental stages, yet the NCD burden and its potential implications for host and ecosystem resilience remain poorly characterized across these nations. This study aimed to systematically estimate the burden, temporal trends, and future projections of NCDs from 1990 to 2021 and up to 2035 in G20 countries.

**Methods:**

Using data from the Global Burden of Disease Study 2021 (GBD2021), we analyzed mortality and years of life lost (YLL) attributable to NCDs among G20 states, stratified by age, sex, and country. Temporal trends were assessed using joinpoint regression to identify inflection points in annual percentage changes. Socioeconomic health inequalities were quantified using the slope index of inequality (SII) and concentration index. Future burden trajectories to 2035 were projected using Bayesian age-period-cohort (BAPC) modeling.

**Results:**

Compared to 1990, the burden of the NCDs across G20 countries in 2021 showed a downward trend. However, the age-standardized mortality and YLL rates from NCDs in G20 countries accounting for 72.21 and 62.56% of all-cause mortality and YLL rates, respectively, markedly surpassing global averages. Specific countries including Indonesia, Russia, Saudi Arabia, and South Africa demonstrated persistently higher burdens compared to regional averages. Males had a higher burden than females. When examining level 2 and level 3 causes of NCDs specifically, trends in burden changes varied across different NCDs at the national levels. Cardiovascular diseases remained the leading contributor to premature mortality, though neurological disorders emerged as the fastest-growing threat. Inequality assessments revealed concentrated burdens in low-sociodemographic index (SDI) region among G20 nations. BAPC model predictions suggested that the burden of NCDs will continue to decline in the future.

**Conclusion:**

Despite an overall reduction in NCD burden, G20 countries face persistent sex disparities and divergent trends between dominant cardiovascular diseases and rapidly rising neurological disorders. These patterns reflect imbalances in host resilience, metabolic stability, and population-level ecosystem health. Targeted interventions are needed to strengthen cardiovascular prevention, enhance neurological care, and mitigate socioeconomic inequalities, with potential implications for restoring host–microbe symbiosis and improving population resilience.

## 1 Introduction

Ecosystem stability and resilience rely on complex symbiotic interaction networks. From soil and aquatic environments to host-associated niches such as the gut and reproductive tract, microbial communities regulate functional homeostasis, stress tolerance, and post-disturbance recovery of ecosystems through mutualism, metabolic exchange, and signal transduction. As the most complex microecological unit in the human body, the host-microbe symbiosis system has been confirmed to be closely linked to the occurrence and development of multiple non-communicable diseases (NCDs) when its structure and function are disrupted (Manzanera, 2025). The epidemic trend and disease burden of NCDs can essentially be regarded as a direct reflection of changes in the resilience of the host-microbe-environment symbiosis system, which constitutes the core background for this study to focus on microbial symbiosis and ecosystem resilience and systematically analyze the burden of NCDs in the Group of Twenty (G20) countries (Li et al., 2022).

NCDs, mainly including cardiovascular diseases, malignant tumors, chronic respiratory diseases (CRDs), and diabetes, have become the primary threat to global population health. In 2021, they caused 43 million deaths (accounting for 75% of non-pandemic-related mortality), representing a continuous increase from 41.1 million deaths in 2017 (accounting for 73.5% of total deaths). Their high incidence, high disability rate, and high mortality not only impose a heavy burden on patients’ families but also exert a significant impact on the medical and health systems and socio-economic development of various countries (Martinez et al., 2020; WHO, 2024; FDI World Dental Federation, 2025). This disease burden is highly unevenly distributed among different populations and regions: 82% of premature deaths occur in low- and middle-income countries, highlighting significant health inequalities; among adolescents, 38.8% of total deaths in 10–24-year-olds in the European Union are attributed to NCDs (Armocida et al., 2022). In 2019, the NCD-related mortality rate for adolescents aged 10–24 was 23.05 per 100,000 population in the South-East Asia Region and 15.59 per 100,000 population in the Western Pacific Region, respectively, reflecting obvious age-specific differences in disease burden (Wang et al., 2023). Epidemiological analyses show a substantial surge in the NCD burden: currently, 7 out of the top 10 global causes of death are NCDs (accounting for 38% of total deaths and 68% of deaths from the top 10 causes), representing a 75% increase compared to 2000 when only 4 out of the top 10 causes of death were NCDs (Ma et al., 2024). Among these, cardiovascular diseases, malignant tumors, chronic respiratory diseases, and diabetes collectively account for more than 80% of NCD-related deaths, and the occurrence and development of these diseases are all closely related to the structural imbalance and functional disorder of the host–microbe symbiosis system (Martins et al., 2024).

The core characteristic of the resilience of the microbial symbiosis system is “resisting disturbances, adapting to changes, and restoring homeostasis.” The long-term prevalence of NCDs is essentially the result of decreased resilience of the host–microbe symbiosis system: when environmental disturbances (such as changes in dietary structure, environmental pollution, and abuse of antibiotics) break the symbiotic balance of microbial communities, the functions of beneficial symbiotic microorganisms are inhibited, and harmful microorganisms over proliferate. This will regulate the host’s immune response and metabolic homeostasis through mechanisms such as abnormal metabolic exchange and disrupted signal transduction, ultimately inducing NCDs (Shah and Ng, 2023). For example, functional imbalance of the gut microbial community is closely related to the occurrence of cardiovascular diseases and diabetes, while disorders of the host–microbe symbiosis system can also accelerate the progression of malignant tumors. These associations confirm the inherent link between microbial symbiosis and the NCD burden, and also highlight the importance of analyzing the NCD burden from the perspective of microbial symbiosis (Li and Guo, 2023; Abdulrahim et al., 2025). The Sustainable Development Goals (SDGs) in the 2030 Agenda for Sustainable Development have identified NCDs as a core challenge to sustainable development and explicitly set the target of reducing NCD-related mortality by one-third by 2030. Therefore, assessing the mortality patterns and epidemic trends of NCDs, combined with their inherent association with the resilience of the host–microbe symbiosis system, is crucial for formulating scientific and reasonable government health policies (NCD Countdown 2030 Collaborators, 2020; WHO, 2024; GBD 2021 Diabetes Collaborators, 2023).

As the core force in global economic and health governance, the Group of Twenty (G20) includes both developed and developing countries, accounting for more than 60% of the global population. The epidemic characteristics and changes in the NCD burden of G20 countries not only reflect the level of public health governance in the region but also reflect the overall situation of the resilience of the global microecological symbiosis system—differences in environmental factors and lifestyles among different countries can directly affect the host–microbe symbiotic balance, thereby regulating the incidence trends and burden patterns of NCDs (Zarocostas, 2024). Over the past three decades, the global health landscape has undergone profound changes, and the changes in the disease spectrum of G20 countries are particularly typical. In the early 1990s, the main causes of death in G20 countries were mainly infectious diseases, maternal and perinatal conditions, and nutritional deficiencies; with the economic development of various countries, improved access to medical care, and changes in lifestyle factors (which can directly change the mode of host–microbe interaction), NCDs have gradually replaced infectious diseases as the primary health threat; by the early 2000s, cardiovascular diseases, cancers, and respiratory diseases had become the main causes of death in G20 countries (Zhang et al., 2026). This transition is closely related to changes in host resilience and microbial community stability at the population level, and is essentially an external manifestation of the evolution of the resilience of the host-microbe-environment symbiosis system.

As the world’s most authoritative disease burden research project, the Global Burden of Disease Study (GBD) 2021 dataset includes core indicators such as the incidence, mortality, and years of life lost (YLL) of NCDs in countries around the world, providing comprehensive data support for analyzing the temporal trends, regional differences, and influencing factors of NCDs. Based on GBD 2021 data, this study first comprehensively summarizes the trends in NCD mortality and YLL, changes in the disease spectrum, and shifts in burden distribution in the world, China, and other G20 countries over the past 30 years, and predicts changes by 2035. More importantly, closely adhering to the core theme of “microbial symbiosis supporting ecosystem resilience,” this study attempts to infer the evolutionary characteristics of the resilience of the host-microbe symbiosis system through changes in the NCD burden, and reveal the core role of microbial symbiosis mechanisms in NCD prevention and control. Tracking the causes and modes of NCD-related deaths not only helps us understand the operation of health systems and the areas that need key support but also provides a new perspective for clarifying the association between host-microbe symbiosis and ecosystem resilience. By analyzing these trends, we hope to clarify their implications for public health policies and practices, ultimately helping to better understand how to improve health outcomes in these diverse countries, and provide scientific support for global NCD prevention and control by maintaining the host-microbe symbiotic balance and enhancing ecosystem resilience.

## 2 Materials and methods

### 2.1 Overview

The GBD, coordinated by the Institute for Health Metrics and Evaluation, 2021 (IHME), is a multinational collaborative initiative spanning 204 countries and territories (1990–2021) to systematically quantify health losses across populations globally. GBD 2021 estimates the burden of 371 diseases and injuries, 88 risk factors, and associated health metrics—including mortality, YLL, and disability-adjusted life years —stratified by age, sex, and sociodemographic index (SDI). The study integrates multisource data from census records, disease registries, hospitalization databases, and systematic literature reviews, standardized through statistical tools such as Bayesian meta-regression (DisMod-MR 2.1), Cause of Death Ensemble Modeling (CODEm), and spatiotemporal Gaussian process regression to ensure cross-regional and temporal comparability. The comprehensive data collection protocols and analytical frameworks implemented in GBD 2021 have been systematically described in prior methodological articles.

We conducted a secondary analysis on the burden of NCDs in G20 countries, extracting age-standardized mortality rates (ASMR) and YLL data from 1990 to 2021. We focused on the G20 countries because they collectively represent 85% of global GDP and 60% of the world’s population, span the full SDI spectrum (from high- to low-SDI economies), and exhibit substantial intra-group heterogeneity in health burdens. ASMR and YLL rates were directly extracted from the GBD 2021 results, which were calculated using the GBD 2021 Standard World Population as the reference standard. This standardization process was implemented using the GBD age_recal () and GBD rei_age_recal () functions.

### 2.2 Data source and definitions

The GBD 2021 categorized causes of death or injury into four distinct levels. level 1 encompassed three major groups: communicable, maternal, neonatal, and nutritional conditions; NCDs; and injuries. Our attention in this study will exclusively be directed toward identifying the causes of NCDs. In this study, we conducted an exclusive analysis of mortality and YLL due to level 1 total NCDs, 12 level 2 NCDs causes, and 101 level 3 NCDs causes by sex, age, location, SDI, and year.

YLL is a metric used to quantify the loss of life years due to deaths occurring earlier than the standard life expectancy as a result of a specific disease or condition. YLL = the number of deaths from NCDs × the standard remaining life expectancy at the time of death. SDI, developed by GBD, summarizes health-related socio-economic conditions. An SDI of 0 indicates minimal health-related development, while 1 signifies maximum. Countries were categorized into five SDI levels according to the SDI values: low, low-middle, middle, high-middle, and high. For the analysis, patients were categorized into 20 age groups (< 5, 5–9, 10–14, 15–19, 20–24, 25–29, 30–34, 35–39, 40–44, 45–49, 50–54, 55–59, 60–64, 65–69, 70–74, 75–79, 80–84, 85–89, 90–94, 95 + years).

### 2.3 Statistical analyses

Both the rate and the number of deaths and YLL cases are used to assess the burden of NCDs. GBD 2021 estimated disease burdens using counts and age-standardized rates (per 100,000 population) derived from 500 computational simulations (reduced from 1,000 in prior cycles without compromising accuracy), with uncertainty intervals (95% UI: 2.5th–97.5th percentiles) integrated throughout modeling stages.

Temporal trends in ASMR and YLL for NCDs across China and G20 nations (1990–2021) were analyzed through Joinpoint regression analysis, identifying significant inflection points in disease burden trajectories. Health disparities were quantified using dual metrics: the Slope Index of Inequality (SII) assessed absolute socioeconomic gradients, while the relative concentration index measured proportional burden distribution patterns. A Bayesian age-period-cohort (BAPC) framework was implemented to project future NCD burden trajectories, with *P* < 0.05 was considered statistically significant. All analyses and visualizations were performed using R Software (version 4.3.2), with the easy GBDR package serving as the core tool for statistical analyses. Methodological specifications are detailed in subsequent sections.

#### 2.3.1 Join point regression analysis

We employed the Joinpoint regression model (Joinpoint Regression Program 5.0.2, National Cancer Institute, United States) to systematically evaluate the long-term dynamic trends of ASMR and YLL rates for NCDs in China and G20 countries from 1990 to 2021. The age-standardized indicators were first subjected to natural logarithmic transformation (ln(y) = β × x + constant, where y represents the standardized rate, x denotes the calendar year, and β is the regression coefficient) to conform to the distribution assumptions of the log-linear model. The model utilized the Grid Search Method to explore all possible combinations of joinpoints, coupled with the Monte Carlo Permutation Test (1,000 repetitions) to identify the optimal number of joinpoints (up to a maximum of 5), thereby balancing model complexity and goodness-of-fit. The final model was validated using the Bayesian Information Criterion to ensure statistical robustness. For trend quantification, the annual percentage change (APC, calculated as APC = (eβ-1) × 100%) and its 95% confidence interval (95% CI) were computed for each segment to determine the significance of local trends (a significant change was defined as a 95% CI excluding zero). The average annual percentage change (AAPC), derived by weighting APCs across segments according to their temporal spans, was used to characterize the overall trend direction during the entire study period (an AAPC > 0 indicated an upward trend, an AAPC < 0 denoted a downward trend, and a 95% CI overlapping zero suggested a stable trend). All methodological procedures strictly adhered to the standardized framework proposed by Kim et al. (2000).

#### 2.3.2 Cross-country inequality analysis

This study employed two complementary inequality metrics—the SII and the relative concentration index—to quantify both absolute and relative inequalities in the burden of NCDs (Cao et al., 2023). The SII was utilized to measure the absolute health disparities across countries with varying SDI levels. The calculation of SII was based on a robust regression model, which mitigates estimation bias induced by data heterogeneity and extreme values by reducing sensitivity to outliers, thereby enhancing the accuracy of health inequality characterization. Specifically, the model regressed national NCD burden against the midpoint of the cumulative population distribution ranked by SDI. An SII value of zero indicates no association between SDI and NCD burden. Positive SII values signify a higher burden in high-SDI countries, whereas negative values reflect a disproportionate burden in low-SDI countries. The concentration index was applied to evaluate proportional disparities in disease burden across the SDI gradient. This involved plotting Lorenz curves by aligning the cumulative proportions of deaths and YLL (vertical axis) with the cumulative population distribution ranked by SDI (horizontal axis). The concentration index, calculated through numerical integration of the area under the Lorenz curve (ranging from −1 to 1), provides the following interpretations: Negative values (curve above the line of equality) indicate concentration of disease burden in low-SDI countries. Zero value (curve coinciding with the equality line) represents an equitable distribution. Positive values (curve below the equality line) signal a higher burden in high-SDI regions. The magnitude of the index reflects the degree of inequality within socioeconomic stratification. In practice absolute values for the concentration index will rarely exceed 0.5, and a value of 0.2–0.3 is considered to represent a reasonably high level of relative inequality. Together, these metrics establish a comprehensive evaluation framework through a dual-dimensional integration of absolute disparities and relative distributional patterns.

#### 2.3.3 Bayesian age-period-cohort (BAPC) analysis

The BAPC model was applied to project NCDs burden across G20 countries (2022–2035), integrating age, period, and cohort effects. Combining generalized linear models with Bayesian inference, the framework employs second-order random walk priors and integrated nested Laplace approximation to smooth temporal fluctuations and compute posterior distributions efficiently, over-coming convergence limitations of traditional Markov chain Monte Carlo methods (Barati et al., 2024; Yuan et al., 2025). Using GBD 1990–2021 data and IHME population projections, the R BAPC package generated age-standardized estimates for mortality and YLL with 95% UIs, enabling identification of high-risk subgroups and policy-relevant trends. The second-order random-integrated nested Laplace approximation integration effectively minimized estimation errors from sparse data and demographic shifts, demonstrating utility for diseases with complex temporal dependencies.

The BAPC model is a robust analytical framework designed to disentangle interdependent age, period, and cohort effects on population health outcomes. By integrating generalized linear models with Bayesian inference, it employs second-order random walk priors to smooth temporal fluctuations and address collinearity inherent in age-period-cohort analyses. The model leverages the integrated nested Laplace approximation to efficiently compute posterior distributions, overcoming computational bottlenecks of traditional Markov chain Monte Carlo methods while quantifying uncertainty through 95% UIs (GBD 2021 Diabetes Collaborators, 2023). Applied to NCDs burden projections across G20 countries (2022–2035), the BAPC framework synthesizes prior epidemiological evidence from the GBD 1990–2021 datasets and IHME population projections, generating age-standardized estimates of mortality and YLL. This approach not only identifies high-risk demographic subgroups but also elucidates policy-relevant temporal trends, demonstrating particular utility in analyzing diseases with complex temporal dependencies and sparse data challenges. Through its capacity to incorporate prior knowledge and rigorously model uncertainty, the BAPC model advances precision in forecasting long-term health burdens and informing targeted interventions. The BAPC code was provided in Supplementary Table 1.

## 3 Results

### 3.1 Burden of NCDs from 1990 to 2021

In 2021, the number of NCD-associated deaths reached 4.4 million (95% UI, 4.2–4.6) in global, 3.2 million (3.1–3.4) in G20 countries, and 1.1 million (0.9–1.2) in China (Supplementary Table 2). The age-standardized mortality rate from NCDs were 529.68 (506.58–554.52) per 100,000 population in global, 506.19 (481.23–535.00) per 100,000 population in G20 countries, and 575.55 (493.41–656.87) per 100,000 population in China, accounting for 64.49, 72.21, and 90.99% of all-cause mortality, respectively. The age-standardized mortality rate from NCDs in China were about 1.09 times and 1.14 times higher than those in global and G20 countries, respectively. At the national level, the highest NCD-related death rates were in Indonesia (738.54 per 100,000 population, 95% UI: 624.27–825.80), Russian Federation (670.30 per 100,000 population, 95% UI: 628.64–711.01), and Saudi Arabia (658.74 per 100,000 population, 95% UI: 572.99–753.79). Whereas the lowest rates were in Japan (262.55 per 100,000 population, 95% UI: 259.50–267.20]), Republic of Korea (295.46 per 100,000 population, 95% UI: 287.40–306.10]), and France (302.96 per 100,000 population, 95% UI: 298.93–308.10), shown in Table 1.

**TABLE 1:** Age-standardized mortality rate and YLL rate due to NCDs in Global and G20 countries, 1990–2021.

Countries	Mortality rate per 100,000 population			YLL rate per 100,000 population		
	1990	2021	Percentage change, 1990–2021	1990	2021	Percentage change, 1990–2021
Global	734.17 (711.90–753.86)	529.68 (506.58–554.52)	−27.85% (−31.39 to −23.92)	17353.86 (16593.95–17983.53)	11954.10 (11322.51–12588.20)	−31.12% (−35.17 to −26.79)
G20	723.73 (698.85) 7474.24	506.19 (481.23–535.00)	−30.06% (−34.07 to −25.17)	16764.34 (16032.17–17425.52)	11018.00 (10459.47–11707.23)	−34.28% (−38.46 to −28.95)
Argentina	689.44 (685.78–694.27)	436.48 (425.81–447.09)	−36.69% (−38.21 to −35.19)	15846.70 (15736.45–15969.42)	9606.98 (9317.23–9911.47	−48.11% (−48.73 to −47.44)
Australia	313.38 (311.42–316.80)	575.19 (571.83–578.83)	−45.52% (−45.95 to −45.04)	11989.88 (11915.85–12072.05)	6221.80 (6160.17–6287.94)	−44.64% (−45.43 to −43.88)
Brazil	662.04 (656.68–669.10)	440.61 (430.65–450.66)	−33.45% (−34.90 to −32.08)	15686.95 (15474.15–15892.27)	10401.45 (10152.75–10653.44)	−21.88% (−27.07 to −16.64)
Canada	527.26 (524.07–531.33)	324.85 (320.32–329.82)	−38.39% (−39.26 to −37.45)	11203.65 (11135.79–11272.49)	6814.00 (6684.46–6952.34)	−39.38% (−41.30 to −37.40)
China	967.52 (881.18–1054.20)	575.55 (493.41–656.87)	−40.51% (−50.15 to −28.52)	20897.32 (18950.06–23007.60)	11020.86 (9359.46–12709.55)	−39.18% (−40.36 to −37.90)
European Union	655.31 (652.89–659.01)	387.13 (378.82–394.80)	−40.92% (−42.16 to −39.83)	14012.91 (13968.49–14065.07)	7749.92 (7536.75–7957.76)	−22.20% (−23.15 to −21.18)
France	518.83 (515.28–524.28)	302.96 (298.93–308.10)	−41.61% (−42.31 to −40.87)	11047.86 (10983.03–11134.08)	6225.06 (6118.24–6337.85)	−42.24% (−42.85 to −41.65)
Germany	654.74 (651.87–658.10)	387.67 (384.26–392.06)	−40.79% (−41.26 to −40.27)	13730.41 (13664.20–13794.91)	7591.91 (7501.51–7680.51)	−47.26% (−56.48 to −35.73)
India	662.74 (579.20–725.34)	603.13 (558.32–655.56)	−8.99% (−19.26 to 3.38)	16965.44 (15103.75–18323.53)	13831.18 (12785.07–14996.93)	−14.84% (−23.15 to −5.55)
Indonesia	683.76 (589.80–760.25)	738.54 (624.27–825.80)	8.01% (−9.46 to 26.12)	17359.47 (15219.96–18982.23)	16330.52 (13869.67–18647.48)	−43.65% (−44.60 to −42.60)
Italy	575.64 (572.83–579.60)	331.13 (328.02–334.66)	−42.48% (−42.98 to −42.03)	11807.76 (11756.44–11867.04)	6136.14 (6062.28–6204.61)	−66.85% (−67.90 to −63.65)
Japan	439.15 (435.49–45.23)	262.55 (259.50–267.20)	−40.21% (−40.66 to −39.76)	8988.79 (8930.46–9065.86)	5191.75 (5135.92–5263.18)	−44.69% (−46.19 to −43.27)
Mexico	629.29 (625.45–634.83)	523.34 (473.99–576.61)	−16.84% (−24.88 to −8.43)	14500.18 (14331.98–14692.03)	12348.29 (11139.47–13660.74)	−33.69% (−35.45 to −31.89)
Republic of Korea	776.52 (750.12–788.15)	295.46 (287.40–306.10)	−61.95% (−62.95 to −59.33)	16584.75 (15747.21–16911.06)	5497.85 (5346.19–5750.17)	−44.71% (−45.37 to −44.06)
Russian Federation	872.10 (869.92–874.27)	670.30 (628.64–711.01)	−23.14% (−28.02 to −18.47)	19496.36 (19437.26–19552.31)	15230.95 (14211.01–16241.09)	−48.03% (−48.72 to −47.40)
Saudi Arabia	815.77 (670.48–962.50)	658.74 (572.99–753.79)	−19.25% (−34.23 to 1.79)	18944.08 (15156.37–22584.20)	14088.60 (11888.33–16609.02)	−18.47% (−26.91 to −8.31)
South Africa	557.18 (524.71–586.42)	647.63 (620.83–674.37)	16.23% (9.63 to 23.47)	13769.97 (13047.63–14422.75)	14540.45 (13833.16–15280.52)	5.60% (−0.92 to 12.60)
Turkey	839.92 (777.72–904.41)	543.62 (464.15–628.18)	−35.28% (−45.84 to −24.33)	21167.70 (18902.90–23311.58)	11063.29 (9399.60–12876.22)	−25.63% (−41.00 to −3.56)
United Kingdom	634.87 (631.44–639.93)	366.65 (361.25–372.20)	−42.25% (−43.00 to −41.57)	13470.14 (13404.70–13538.27)	7457.01 (7348.39–7561.86)	−5.93% (−20.96 to 9.89)
United States of America	574.21 (572.01–577.72)	442.80 (437.88–448.16)	−22.89% (−23.64 to −22.07)	12792.47 (12751.20–12844.95)	9952.83 (9830.80–10079.60)	−47.74% (−56.61 to −37.77)

**FIGURE 1:**
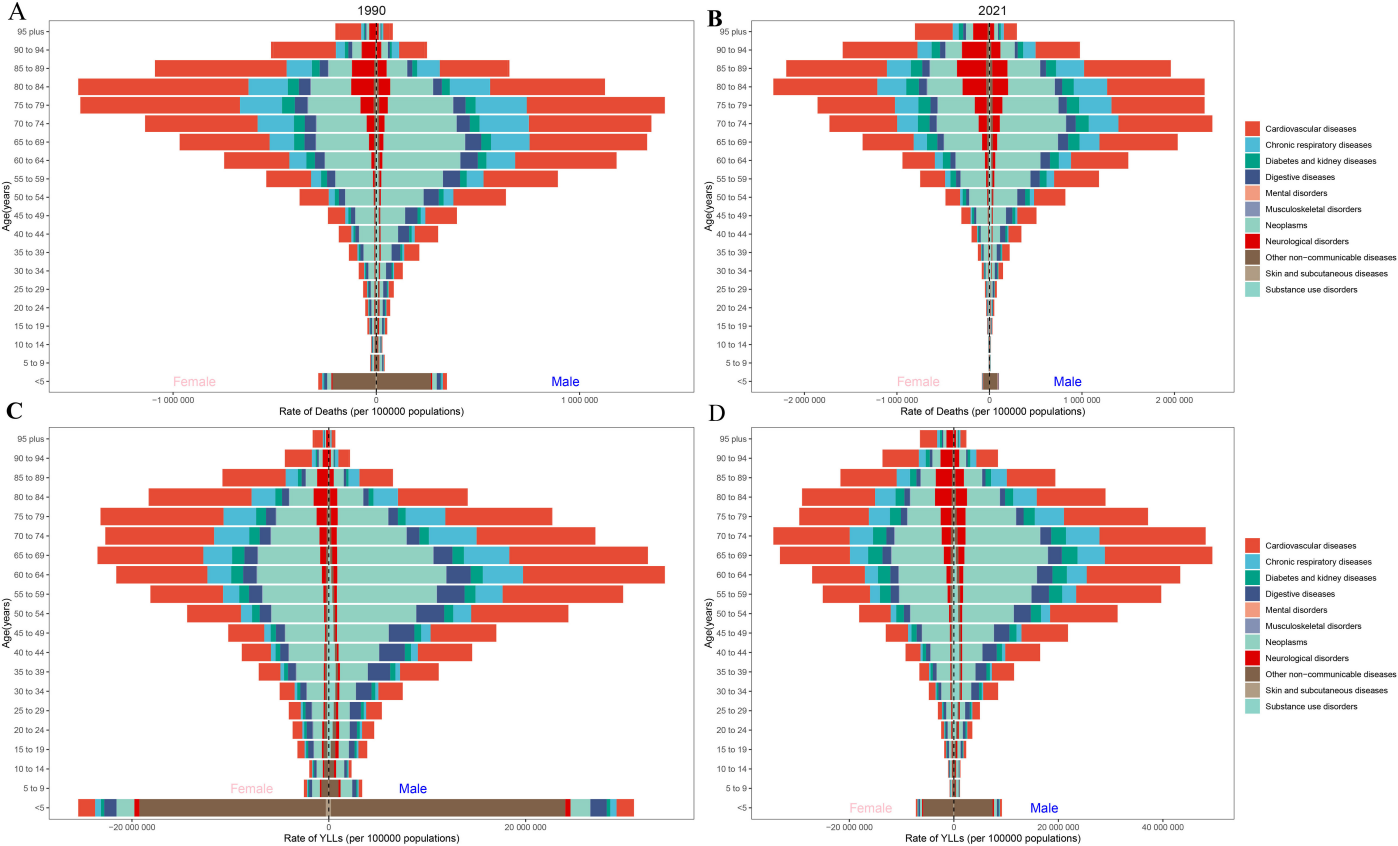
Gender and age disparities of mortality rate and YLL rate due to NCDs in G20 countries, 1990–2021. **(A,B)** Mortality rate. **(C,D)** YLL rate. The *X*-axis represents the rate per 100,000 population, the *Y*-axis represents age groups. Color coding indicates categories of NCDs as shown in the legend.

In 2021, age-standardized mortality and YLL rates for males consistently exceeded those for females across global, G20, and Chinese populations (Table 2), with cardiovascular diseases remaining the predominant cause of mortality. Globally, NCDs demonstrated an age-dependent mortality gradient: the highest rate was observed in individuals aged ≥ 95 years (22,955.87, 95% UI: 22,265.92–23,778.92), while the lowest occurred in the 5∼9-year age group (11.38, 95% UI: 9.94–12.77). A notable pattern emerged in children under 5 years, who exhibited elevated NCD mortality (97.14, 95% UI: 81.13–116.40), surpassing rates in the 30∼34 age group (63.13, 95% UI: 59.64–66.64). For individuals above 35 years, NCD mortality displayed a progressive decline with advancing age. Comparative analysis revealed parallel age-specific NCD mortality patterns between G20 nations and China relative to global trends. Within G20 countries, the age-standardized YLL rate in the under-5 cohort (8,666.92, 95% UI: 7,241.48–10,381.53) significantly exceeded those observed in the 25∼29 age group and younger cohorts (Table 2). In G20 countries, age-standardized mortality and YLL rates among females demonstrated substantial declines of 32.25% (95%UI: −37.55 to −26.46) and 36.55% (95% UI: −41.97 to −30.30), respectively, exceeding reductions observed in males. Age-stratified analysis revealed the most pronounced mortality decrease in the under-5 cohort (−65.61%, 95% UI: −73.29 to −46.50), contrasting with the smallest reduction in the ≥ 95-year age group (−27.00%, 95% UI: −18.98 to −14.65), shown in Figure 1. Notably, China exhibited substantially greater improvements in pediatric health outcomes, with under-5 mortality and YLL rates declining by 84.32% (95% UI: −88.71 to −75.54) and 84.28% (95% UI: −88.68 to-75.46), respectively.

**TABLE 2:** Age-standardized mortality rate and YLL rate due to NCDs in Global and G20 countries by sex and age, from 1990 to 2021.

	Mortality rate per 100,000 population			YLL rate per 100,000 population		
	1990	2021	Percentage change, 1990–2021	1990	2021	Percentage change, 1990–2021
Global						
Gender/sex						
Male	864.82 (829.40–900.07)	634.51 (597.92–673.83)	−26.63% (−31.72 to −21.16)	20474.55 (19423.0221449.12 −)	14292.03 (13368.55–15174.28)	−30.20% (−35.51 to −24.78)
Female	630.02 (604.01–652.67)	443.60 (420.09–467.95)	−29.59% (−33.75 to −24.91)	14605.04 (13786.03–15286.11)	9859.90 (9269.81–10468.59)	−32.49% (−37.35 to −27.28)
Age (years)						
< 5	209.62 (150.67–251.82)	97.14 (81.13–116.40)	−53.66% (−63.02 to −33.61)	18695.97 (13440.82–22456.87)	8666.92 (7241.48–10381.53)	−53.64% (−62.99 to −33.59)
5∼9	22.27 (19.02–24.97)	11.38 (9.94–12.77)	−48.90% (−55.34 to −37.89)	1846.03 (1576.42–2069.64)	942.65 (823.19–1057.40)	−48.94% (−55.37 to −37.93)
10∼14	18.47 (16.68–19.94)	12.27 (11.09–13.38)	−33.60% (−38.82 to −26.68)	1432.64 (1293.59–1546.21)	950.68 (859.52–1036.82)	−33.64% (−38.85 to −26.72)
15∼19	29.98 (27.90–31.94)	22.23 (20.37–24.11)	−25.86% (−31.45 to −20.48)	2175.04 (2024.05–2317.24)	1612.25 (1477.14–1748.60)	−25.88% (−31.47 to −20.5)
20∼24	37.88 (35.51–40.05)	30.88 (28.64–33.00)	−18.49% (−24.45 to −12.48)	2563.42 (2402.84–2709.91)	2088.44 (1937.17–2232.24)	−18.53% (−24.49 to −12.52)
25∼29	50.79 (48.24–53.17)	41.00 (38.54–43.53)	−19.28% (−24.13 to −14.26)	3187.16 (3027.01–3336.66)	2569.92 (2415.93–2728.63)	−19.37% (−24.21 to −14.34)
30∼34	80.37 (76.84–83.79)	63.13 (59.64–66.64)	−21.45% (−26.12 to −16.8)	4638.33 (4434.17–4835.27)	3643.45 (3441.53–3845.82)	−21.45% (−26.12 to −16.81)
35∼39	133.92 (128.39–140.06)	98.90 (93.40–104.60)	−26.15% (−30.71 to −21.26)	7067.70 (6776.09–7392.18)	5220.73 (4930.14–5521.68)	−26.13% (−30.7 to −21.24)
40∼44	230.53 (221.65–241.16)	170.01 (160.43–179.85)	−26.25% (−30.97 to −21.33)	11038.41 (10613.50–11547.53)	8135.88 (7677.26–8606.73)	−26.29% (−31.01 to −21.38)
45∼49	375.89 (360.90–391.80)	263.51 (247.78–279.70)	−29.9% (−34.50 to −24.82)	16133.38 (15489.92–16816.92)	11321.43 (10646.09–12016.83)	−29.83% (−34.43 to −24.75)
50∼54	652.65 (627.37–679.46)	432.55 (406.37–460.94)	−33.72% (−38.24 to −28.56)	24913.84 (23950.12–25936.70)	16518.53 (15519.16–17600.74)	−33.7% (−38.21 to −28.54)
55∼59	1030.61 (989.71–1075.74)	699.97 (659.30–745.50)	−32.08% (−36.59 to −26.75)	34501.36 (33131.08–36014.00)	23461.40 (22097.19–24988.84)	−32% (−36.52 to −26.66)
60∼64	1609.27 (1553.21–1667.10)	1104.06 (1048.17–1165.27)	−31.39% (−35.34 to −27.04)	46458.10 (44838.35–48128.91)	31876.90 (30264.42–33642.70)	−31.39% (−35.33 to −27.03)
65∼69	2445.61 (2355.72–2530.16)	1697.24 (1608.85–1796.72)	−30.60% (−34.78 to −25.88)	59474.39 (57288.37–61527.58)	41258.19 (39110.94–43672.63)	−30.63% (−34.8 to −25.92)
70∼74	3828.11 (3694.77–3962.85)	2665.27 (2531.31–2818.73)	−30.38% (−34.48 to −25.67)	76536.73 (73866.64–79233.26)	53323.20 (50646.72–56391.09)	−30.33 (−34.43 to −25.63)
75∼79	5939.01 (5762.28–6098.12)	4181.78 (3989.21–4402.89)	−29.59% (−33.30 to −25.43)	94821.06 (91991.17–97370.51)	66752.64 (63674.90–70281.95)	−29.6% (−33.31 to −25.45)
80∼84	9143.48 (8928.83–9343.49)	6875.90 (6596.41–7178.49)	−24.80% (−28.08 to −21.18)	114398.96 (111710.73–116903.61)	85727.16 (82246.65–89495.22)	−25.06% (−28.33 to −21.46)
85∼89	14245.46 (13975.43–14520.01)	11240.06 (10834.59–11654.66)	−21.10% (−24.09 to −17.76)	141566.50 (138877.34–144300.51)	111251.78 (107238.74–115356.07)	−21.41% (−24.4 to −18.08)
90∼94	20901.36 (20506.99–21378.02)	17030.34 (16493.42–17592.48)	−18.52% (−20.95 to −15.92)	180234.90 (176833.96–184346.79)	146753.45 (142119.98–151592.00)	−18.58% (−21.01 to −15.97)
95 +	27701.18 (27088.06–28663.17)	22955.87 (22265.92–23778.92)	−17.13% (−19.19 to −14.93)	225449.31 (220460.39–233245.62)	185344.03 (179758.18–191975.41)	−17.79% (−19.85 to −15.58)
G20 countries						
Gender/Sex						
Male	870.45 (827.79–912.46)	620.45 (576.93–668.57)	−28.72% (−34.68 to-21.58)	20140.68 (19043.01–21258.67)	13508.77 (12554.49–14595.14)	−32.93% (−38.91 to −25.76)
Female	611.79 (582.67–638.70)	413.86 (387.18–443.33)	−32.25% (−37.55 to −26.46)	13855.62 (13047.67–14613.46)	8790.74 (8191.77–9402.04)	−36.55% (−41.97 to −30.30)
Age (years)						
< 5	170.75 (132.54–203.59)	58.72 (48.30–71.34)	−65.61% (−73.29 to 46.50)	15230.65 (11821.93–18155.97)	5249.15 (4318.32–6379.16)	−65.54% (−73.24 to −46.38)
5∼9	19.66 (17.24–21.78)	6.89 (6.14–7.76)	−64.97% (−69.09 to −58.16)	1629.35 (1428.85–1804.81)	570.23 (508.22–642.16)	−65.00% (−69.12 to −58.19)
10∼14	16.16 (14.57–17.44)	8.64 (8.00–9.29)	−46.51% (−50.44% to −41.00%)	1252.37 (1129.34–1352.27)	669.24 (619.53–719.29)	−46.56% (−50.49 to −41.05)
15∼19	27.79 (26.00–29.69)	16.88 (15.84–17.99)	−39.27% (−44.19% to −33.83)	2015.30 (1885.03–2152.89)	1223.20 (1147.72–1303.76)	−39.30 (−44.23 to −333.87)
20∼24	35.44 (33.24–37.74)	25.62 (24.26–27.27)	−27.69% (−33.99% to −20.54)	2397.93 (2249.50–2553.73)	1732.40 (1640.01–1843.53)	−27.75% (−34.05 to −20.60)
25∼29	48.08 (45.67–50.83)	36.30 (34.52–38.42)	−24.50% (−30.71 to −18.24)	3017.30 (2865.54–3189.54)	2274.27 (2162.73–2407.22)	−24.63% (−30.83 to −18.38)
30∼34	78.57 (75.04–82.20)	59.31 (56.16–62.88)	−24.51% (−30.09 to 18.48)	4531.66 (4328.05–4740.68)	3421.07 (3239.33–3626.97)	−24.51% (−30.09 to −18.49)
35∼39	132.60 (126.07–139.81)	94.23 (89.18–99.97)	−28.94% (−33.84 to −22.74)	6997.42 (6653.19–7378.03)	4973.71 (4706.94–5276.92)	−28.92 (−33.83 to −22.72)
40∼44	226.65 (215.44–238.24)	163.08 (153.72–174.08)	−28.05% (−33.33 to −21.48)	10852.65 (10315.61–11407.62)	7801.63 (7354.09–8327.64)	−28.11% (−33.40 to −21.55)
45∼49	246.91 (231.75–265.33)	366.86 (349.37–384.72)	−32.70% (−37.75 to 26.02)	15745.27 (14993.99–16512.11)	10603.49 (9953.99–11391.97)	−32.66% (−37.71 to −25.98)
50∼54	637.35 (603.54–670.06)	403.49 (374.46–436.86)	−36.69% (−42.10 to −29.83)	24321.93 (23033.01–25568.76)	15399.23 (14291.97–16672.05)	−36.69% (−42.09 to −29.83)
55∼59	1016.04 (961.88–1069.92)	663.09 (617.22–715.74)	−34.74% (−40.32 to −27.79)	34010.66 (32195.78–35816.30)	22221.56 (20682.80–23987.74)	−34.66% (−40.26 to −27.70)
60∼64	1567.05 (1492.93–1635.69)	1038.44 (979.15–1108.20)	−33.73% (−38.58 to −27.78)	45232.72 (43092.36–47214.63)	29963.68 (28256.50–31972.82)	−33.76% (−38.60 to −27.81)
65∼69	2390.21 (2285.74–2493.22)	1606.57 (1505.92–1722.11)	−32.79% (−37.69 to −26.47)	58109.44 (55572.90–60614.92)	39027.57 (36583.75–41830.83)	−32.84% (−37.74 to −26.53)
70∼74	3784.95 (3620.21–3946.52)	2551.15 (2397.47–2728.69)	−32.60% (−37.59 to −26.78)	75655.23 (72359.50–78884.07)	50998.34 (47929.04–54549.28)	−32.59% (−37.57 to −26.77)
75∼79	5869.68 (5666.01–6056.13)	4019.74 (3781.96–4268.83)	−31.52% (−36.05 to 26.54)	93682.28 (90420.10–96667.62)	64132.72 (60335.77–68108.32)	−31.54% (−36.08 to −26.56)
80∼84	9119.22 (8888.90–9356.79)	6649.21 (6322.35–6975.44)	−27.09 (−31.11 to −22.98)	114071.76 (111183.11–117053.15)	82836.16 (78768.98–86898.03)	−27.38% (−31.39 to −23.29)
85∼89	14325.61 (14034.58–14634.92)	11098.95 (10621.42–11564.98)	−22.52% (−26.13 to −18.67)	142335.50 (139434.14–145420.18)	109764.16 (105038.13–114380.55)	−22.88% (−26.48 to −19.04)
90∼94	21248.52 (20869.63–21708.79)	17108.54 (16501.48–17705.98)	−19.48% (−22.28 to 16.54)	183236.58 (179965.01–187205.95)	147422.33 (142186.77–152578.15)	−19.55% (−22.34 to −16.60)
95 +	28484.62 (27969.05–29364.96)	23641.26 (22927.53–24557.83)	−27.00% (−18.98 to −14.65)	232055.14 (227861.04–239214.23)	190793.73 (185037.11–198206.45)	−17.78% (−19.75 to −15.43)
China						
Gender/Sex						
Male	1150.82 (1298.38–1008.16)	765.91 (916.15–634.18)	−33.45% (−48.76 to −16.22)	24369.39 (27919.47–21102.81)	14388.52 (17505.22–11662.69)	−40.96% (−55.44 to −24.25)
Female	837.09 (949.08–732.61)	438.21 (528.47–363.73)	−47.65% (−58.38 to −33.27)	17903.82 (20551.99–15539.57)	8144.77 (9922.76–6700.51)	−54.51% (−64.51 to −41.83)
**Age (years)**			**Age (years)**			
< 5	221.33 (278.79–167.76)	34.7 (43.91–27.7)	−84.32% (−88.71 to −75.54)	19701.80 ( 24808.55–14930.27)	3096.41 (3918.4–2472.62)	−84.28% (−88.68 to −75.46)
5∼9	25.51 (28.56–22.49)	7.92 (9.24–6.78)	−68.97% (−74.18 to −62.27)	2114.99 (2368.08–1864.30)	655.85 (765.35–561.97)	−68.99% (−74.19 to −62.29)
10∼14	18.51 (20.4–16.51)	7.45 (8.37–6.56)	−59.75% (−65.5 to −52.81)	1433.54 (1580.14–1278.50)	576.59 (648.02–507.98)	−59.78% (−65.53 to −52.84)
15∼19	30.01 (33.49–26.65)	12.97 (15.25–11.07)	−56.77% (−64.62 to −46.65)	2175.44 (2427.61–1932.06)	940.05 (1104.71–802.22)	−56.79% (−64.64 to −46.68)
20∼24	34.96 (39.82–30.47)	20.67 (24.5–17.23)	−40.89% (−52.78 to −25.02)	2366.27 (2694.78–2062.48)	1396.52 (1655.51–1164.55)	−40.98% (−52.85 to −25.14)
25∼29	43.31 (48.48–38.31)	26.83 (31.17–22.88)	−38.06% (−48.6 to −23.62)	2721.77 (3046.38–2407.57)	1678.43 (1950.12–1431.36)	−38.33% (−48.83 to −23.96)
30∼34	77.27 (86.69–68.94)	46.25 (53.61–39.17)	−40.14% (−50.65 to −26.19)	4444.57 (4986.44–3965.17)	2663.19 (3086.8–2255.25)	−40.08% (−50.59 to −26.11)
35∼39	139.87 (156.86–123.89)	78.27 (91.78–65.13)	−44.04% (−55 to −29.81)	7381.19 (8277.75–6538.19)	4134.9 (4848.64–3440.62)	−43.98% (−54.95 to −29.74)
40∼44	259.13 (292.74–225.58)	137.21 (163.57–112.23)	−47.05% (−58.59 to −32.13)	12413.91 (14023.4–10807.07)	6557.69 (7818.01–5363.94)	−47.17% (−58.69 to −32.29)
45∼49	389.53 (443.57–335.3)	197.35 (239.39–158.32)	−49.33% (−61.44 to −33.73)	16723.41 (19043.81–14394.65)	8449.54 (10249.52–6778.24)	−49.47% (−61.55 to −33.91)
50∼54	724.32 (824.96–625.28)	343.62 (417.2–275.35)	−52.56% (−64.08 to −37.85)	27604.46 (31440.78–23829.36)	13094.42 (15899.05–10493.13)	−52.56% (−64.09 to −37.85)
55∼59	1152.65 (1316.95–994.79)	542.29 (659.14–434.52)	−52.95% (−64.36 to −38.26)	38632.5 (44139.15–33342.28)	18192.61 (22113.16–14576.9)	−52.91% (−64.32 to −38.2)
60∼64	1761.44 (1989.82–1537.77)	912.44 (1091.84–745.16)	−48.20% (−59.86 to −33.42)	50861.36 (57454.01–44403.16)	26258.51 (31422.4–21444.07)	−48.37% (−60 to −33.64)
65∼69	2837.39 (3172.46–2503.84)	1505.78 (1786.21–1241.21)	−46.93% (−58.17 to −32.7)	68885.52 (77021.99–60784.83)	36501.49 (43303.77–30090.09)	−47.01% (−58.24 to −32.8)
70∼74	5001.29 (5524.18–4474.49)	2776.25 (3245.63–2319.6)	−44.49% (−55.2 to −30.99)	99895.62 (110350.93–89375.89)	55450.19 (64822.91–46327.86)	−44.49% (−55.2 to −31)
75∼79	7933.75 (8695.69–7184.84)	4710.47 (5427.17–3998.38)	−40.63% (−51 to −27.75)	127138.75 (139353.77–115142.83)	75117.26 (86553.43–63761.56)	−40.92% (−51.25 to −28.1)
80∼84	13050.94 (14054.67–12019.71)	8691.2 (9772.46–7579.11)	−33.41% (−42.49 to −21.85)	163588.34 (176163.03–150658.91)	108136.92 (121598.98–94302.31)	−33.90% (−42.91 to −22.42)
85∼89	23035.01 (24606.96–21527.9)	16254.27 (17979.7–14483.47)	−29.44% (−38.29 to −19.1)	229983.68 (245680.99–214944.84)	160858.3 (177932.29–143338.11)	−30.06% (−38.83 to −19.82)
90∼94	36046.93 (38464.28–33961.37)	24919.65 (27495.52–22253.95)	−30.87% (−39.4 to −21.22)	311808.29 (332693.04–293774.31)	215070.01 (237312.83–192053.15)	−31.02% (−39.53 to −21.4)
95 +	42372.75 (44327.87–40175.42)	31250.79 (35249.25–27791.47)	−26.25% (−35.13 to −15.6)	350154.75 (366321.83–331997.98)	254924.04 (287433.82–226790.07)	−27.20% (−35.98 to −16.69)

**FIGURE 2:**
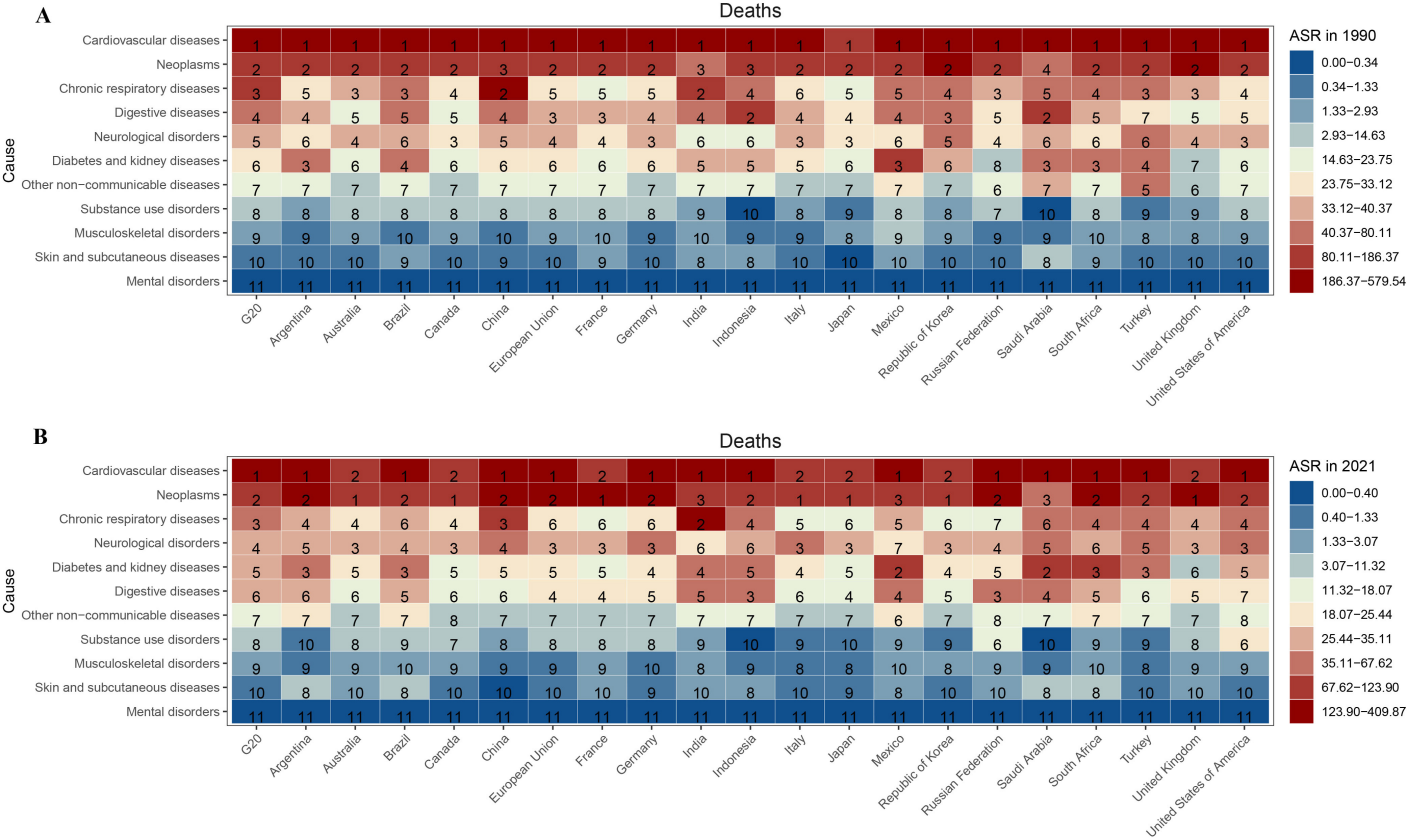
Ranking of ASMR for NCDs across geographic regions in 1990 **(A)** and 2021 **(B)**. The colors in the figure represent rankings from high (red) to low (blue), A chromatic gradient scale (red: highest; blue: lowest) visualizes mortality rankings, with numerical annotations denoting the ordinal positions of 11 types of NCDs in G20 countries.

In 2021, cardiovascular diseases were the leading cause of mortality among second-level NCDs, with ASMRs per 100,000 population estimated at 235.18 (95%UI: 214.64–250.52) globally, 218.13 (95% UI: 195.80–235.27) in G20 countries, and 280.11 (95% UI: 237.90–323.90) in China (Figure 2 and Table 3). Neoplasms were the second most common cause, with mortality rates of 116.49 (95% UI: 107.28–124.69) per 100,000 population globally, 120.39 (95% UI: 109.51–129.94) per 100,000 population in G20 countries, and 137.48 (95% UI: 115.11–163.38) per 100,000 population in China. Chronic respiratory diseases ranked third, with mortality rates of 53.56 (95% UI: 48.46–59.09) per 100,000 population globally, 55.98 (95% UI: 50.04–61.89) per 100,000 population in G20 countries, and 75.81 (95% UI: 61.99–89.88) per 100,000 population in China. In 2021, ischemic heart disease was the leading level 3 cause of death, with ASMR per 100,000 population estimated at 108.73 (95% UI: 99.60–115.38) globally, 99.96 (95% UI: 90.44–106.96) in G20 countries, and stroke were the leading causes in China (138.03, 95% UI: 116.69–160.32) (Supplementary Table 3).

**TABLE 3:** Age-standardized mortality rate and YLL rate from Level 2 causes of NCDs in Global and G20 countries, 1990–2021.

	Mortality rate per 100,000 population			YLL rate per 100,000 population		
	1990	2021	Percentage change, 1990–2021	1990	2021	Percentage change, 1990–2021
Global						
Neoplasms	148.24 (140.18–154.39)	116.49 (107.28–124.69)	−21.42% (−26.55 to −15.42)	3881.31 (3711.65–4040.12)	2860.53 (2684.95–3062.39)	−26.30% (−31.34 to −20.33)
Cardiovascular diseases	358.12 (333.74–372.63)	235.18 (214.64–250.52)	−34.33% (−37.79 to −30.75)	7161.52 (6806.73–7430.69)	4671.63 (4369.38–4957.14)	−34.77% (−38.48 to −30.92)
Chronic respiratory diseases	84.55 (76.19–90.46)	53.56 (48.46–59.09)	−36.66% (−42.64 to −27.11)	1678.78 (1517.02–1793.11)	975.01 (892.86–1069.63)	−41.92% (−47.43 to −32.47)
Digestive diseases	46.77 (42.91–52.10)	29.97 (27.27–32.58)	−35.92% (−45.49 to −26.40)	1396.49 (1290.90–1537.14)	867.81 (807.96–940.75)	−37.86% (−46.11 to −29.19)
Neurological disorders	32.91 (14.36–73.74)	33.22 (15.07–71.70)	0.96% (−3.60 to 7.21)	523.99 (295.54–1009.22)	510.13 (286.60–984.59)	−2.65% (−7.64 to 2.35)
Mental disorders	0.00 (0.00–0.00)	0.00 (0.00–0.00)	−1.73% (−9.99 to 7.26)	0.16 (0.14–0.18)	0.16 (0.14–0.18)	−2.31% (−10.34 to 6.82)
Substance use disorders	3.73 (3.53–3.88)	3.49 (3.21–3.69)	−6.48% (−12.58 to −0.34)	157.24 (148.75–164.03)	146.96 (135.72–155.42)	−6.54% (−12.78 to −0.23)
Diabetes and kidney diseases	33.32 (31.33–35.28)	38.24 (35.38–40.57)	14.79% (7.09 to 21.03)	800.94 (757.50–844.12)	867.84 (813.09–923.63)	8.35% (0.53 to 14.88)
Skin and subcutaneous diseases	1.39 (1.22–1.56)	1.47 (1.34–1.57)	6.46% (−4.97 to 20.20)	39.90 (35.83–43.63)	35.90 (32.88–39.20)	−10.03% (−18.55 to 1.82)
Musculoskeletal disorders	1.55 (1.41–1.66)	1.44 (1.25–1.56)	−7.23% (−15.82 to −0.90)	40.26 (36.44–43.35)	35.01 (30.32–37.83)	−13.04% (−21.74 to −6.63)
Other non-communicable diseases	23.59 (17.90–28.05)	16.60 (15.09–18.59)	−29.60% (−39.42 to −6.03)	1673.26 (1150.32–2068.18)	983.12 (858.21–1150.20)	−41.24% (−51.69 to −13.56)
G20						
Neoplasms	157.84 (148.44–164.85)	120.39 (109.51–129.94)	−23.73% (−29.60 to −17.09)	4105.39 (3897.08–4294.84)	2901.33 (2688.91–3138.27)	−29.33% (−34.92 to −22.50)
Cardiovascular diseases	344.11 (318.51–359.02)	218.13 (195.80–235.27)	−36.61% (−41.06 to −31.76)	6786.04 (6429.08–7073.83)	4262.88 (3960.77–4567.56)	−37.18% (−41.45 to −31.87)
Chronic respiratory diseases	89.28 (80.87–95.97)	55.98 (50.04–61.89)	−37.30% (−44.13 to −27.32)	1737.26 (1568.30–1870.59)	996.93 (911.07–1098.62)	−42.61% (−48.80 to −32.48)
Digestive diseases	42.63 (39.10–47.17)	26.36 (23.12–29.43)	−38.17% (−49.05 to −28.02)	1264.13 (1171.97–1397.51)	741.13 (655.58–833.06)	−41.37% (−51.83 to −31.81)
Neurological disorders	33.89 (14.65–75.86)	33.92 (15.20–73.19)	0.10% (−5.11 to 6.94)	533.16 (297.94–1029.38)	504.45 (276.21–996.53)	−5.39% (−11.59 to 0.28)
Mental disorders	0.00 (0.00–0.00)	0.00 (0.00–0.00)	13.65% (4.28 to 23.95)	0.21 (0.18–0.23)	0.24 (0.20–0.27)	13.52% (4.29 to 23.96)
Substance use disorders	3.86 (3.66–4.05)	4.09 (3.83–4.33)	5.87% (−1.64 to 13.30)	166.51 (157.49–175.45)	177.31 (166.34–187.30)	6.49% (−1.23 to 14.17)
Diabetes and kidney diseases	28.83 (26.95–30.38)	31.80 (29.09–33.81)	10.28% (2.37 to 17.20)	675.71 (637.95–712.85)	692.93 (645.85–735.78)	2.55% (−5.06 to 9.50)
Skin and subcutaneous diseases	1.33 (1.20–1.44)	1.33 (1.20–1.43)	−0.46% (−9.90 to 11.82)	39.98 (36.19–43.51)	32.66 (30.12–35.32)	−18.33% (−26.45 to −7.19)
Musculoskeletal disorders	1.66 (1.51–1.77)	1.48 (1.29–1.63)	−11.04% (−19.69 to −3.66)	42.23 (38.65–45.30)	34.29 (30.50–37.63)	−18.81% (−27.52 to −11.30)
Other non-communicable diseases	20.30 (16.51–23.61)	12.72 (11.56–14.21)	−37.33% (−47.03 to −15.45)	1413.71 (1063.65–1715.78)	673.86 (584.95–793.17)	−52.33% (−61.78 to −26.38)
China						
Neoplasms	185.53 (163.83–208.63)	137.48 (115.11–163.38)	−25.90% (−40.18 to −6.74)	5083.45 (4470.34–5718.28)	3378.14 (2833.19–4028.23)	−33.55% (−46.87 to −15.68)
Cardiovascular diseases	407.72 (361.40–452.12)	280.11 (237.90–323.90)	−31.30% (−42.40 to −17.37)	7698.41 (6808.16–8560.81)	4708.95 (3959.35–5496.60)	−38.83% (−49.35 to −25.07)
Chronic respiratory diseases	239.34 (205.82–265.39)	75.81 (61.99–89.88)	−68.33% (−74.74 to −60.42)	3816.92 (3272.56–4251.87)	1087.99 (888.11–1307.41)	−71.50% (−77.41 to −64.01)
Digestive diseases	42.37 (36.40–48.89)	14.76 (11.89–17.75)	−65.17% (−73.91 to −54.61)	1177.16 (1014.92–1350.74)	360.78 (290.00–431.75)	−69.35% (−77.08 to −59.63)
Neurological disorders	39.59 (16.15–91.48)	37.30 (14.57–88.39)	−5.78% (−19.56 to 11.64)	602.03 (309.99–1223.85)	520.35 (241.22–1185.19)	−13.57% (−30.17 to 2.75)
Mental disorders	0.00 (0.00–0.00)	0.00 (0.00–0.00)	514.01% (232.33 to 1658.05)	0.02 (0.01–0.04)	0.12 (0.07–0.17)	504.15% (223.01 to 1655.07)
Substance use disorders	3.67 (3.14–4.25)	1.67 (1.20–2.11)	−54.52% (−66.62 to −41.43)	163.99 (140.81–190.15)	73.23 (54.32–91.67)	−55.35% (−66.94 to −42.48)
Diabetes and kidney diseases	25.01 (22.30–28.69)	20.13 (16.79–23.71)	−19.52% (−35.81 to −1.22)	610.94 (542.08–695.13)	418.84 (347.75–499.38)	−31.44% (−45.87 to −14.54)
Skin and subcutaneous diseases	1.24 (1.06–1.45)	0.40 (0.33–0.48)	−67.74% (−75.61 to −57.63)	28.47 (24.97–31.99)	6.95 (5.94–8.10)	−75.59% (−80.40 to −69.44)
Musculoskeletal disorders	1.22 (1.00–1.49)	1.08 (0.84–1.35)	−11.17% (−36.78 to 14.21)	37.65 (31.18–45.95)	29.16 (22.81–36.05)	−22.55% (−45.78 to 2.12)
Other non-communicable diseases	21.83 (16.23–27.57)	6.81 (5.86–7.91)	−68.81% (−76.04 to −53.46)	1678.29 (1167.74–2185.62)	436.34 (360.04–527.09)	−74.00% (−80.92 to −56.67)

### 3.2 Jion point regression analysis of temporal trends in NCDs in G20 countries

From 1990 to 2021, the global mortality rate associated with NCDs has declined by 27.85% (−31.39 to −23.92). Among G20 countries, the mortality rate has decreased from 723.73 (698.85–7474.24) to 506.19 (481.23–535.00), with a decreasing trend of 1.15%/year (−1.17 to −1.12). The NCD-related death rate decreased in most countries, especially in Republic of Korea (AAPC: −3.1%, 95% CI: −3.17 to −3.06), Australia (AAPC: −1.94%, 95% CI: −2.0 to −1.86), Italy (AAPC: −1.79%, 95% CI: −1.83 to −1.75), United Kingdom (AAPC: −1.77%, 95% CI: −1.82 to −1.73), and France (AAPC: −1.63%, 95% CI: −1.66 to −1.59). Similarly, China has experienced a 1.69%/year (−1.73 to −1.66) reduction in NCD-related mortality. However, the mortality rates in Indonesia (8.01%, 95% UI: −9.46 to 26.12) and South Africa (16.23%, 95% UI: 9.63 to 23.47) have increased (Table 1).

In 2021, the number of NCD-associated YLL were 99.5 million (95% UI: 94.3–104.62) in global, 67.7 million (95% UI: 64.1–71.) in G20 countries, and 21.1 million (95% UI: 17.7–24.7) in China. The YLL rates from NCDs were 11954.10 (95% UI: 11322.51–12588.20) per 100,000 population in global, 11018.00 (95% UI: 10459.47–11707.23) per 100,000 population in G20 countries, and 11020.86 (95% UI: 9359.46–12709.55) per 100,000 population in China, accounting for 50.36%, 62.56 and 85.48 of all-cause YLL rates, respectively. The countries with the highest rates of YLL due to NCDs were Indonesia (16,330.52, 95%UI: 13869.67–18647.48), the Russian Federation (15230.95, 95%UI: 14211.01–16241.09), and South Africa (14540.45,95% UI: 13833.16–15280.52). Conversely, the countries with the lowest YLL rates were Japan (5191.75, 95% UI: 5135.92–5263.18), Republic of Korea (5497.85, 95% UI: 5346.19–5750.17), and Italy (6136.14, 95% UI: 6062.28–6204.61) (Table 1).

**FIGURE 3:**
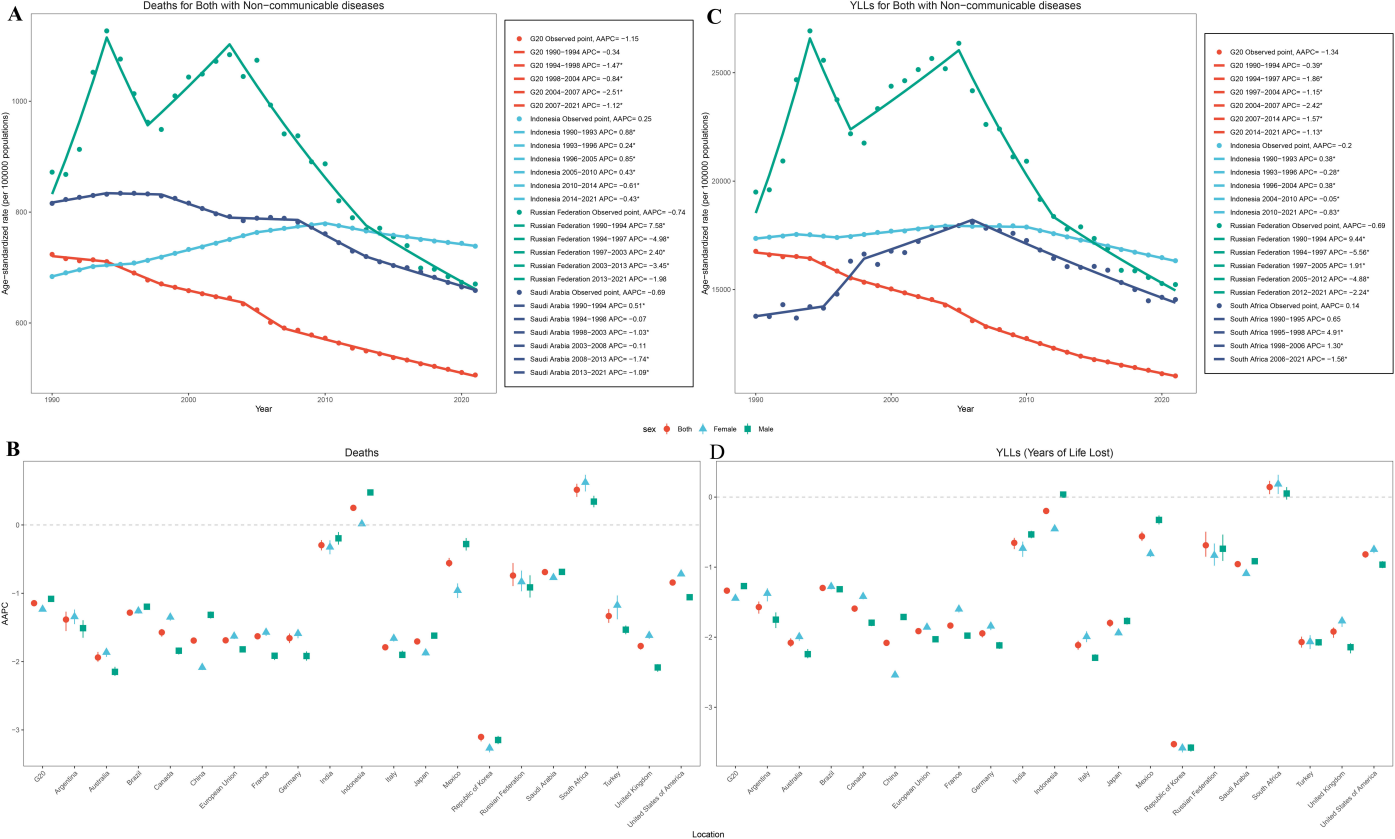
Join point regression analysis of age-standardized mortality rate and YLL rate due to NCDs in G20 countries and countries with the highest rate in 2021, 1990–2021. **(A,B)** Age-standardized mortality rate. **(C,D)** Age-standardized YLL rate. Colors: Red = G20 average; Light blue = Indonesia; Dark green = Russian Federation; Dark blue = Saudi Arabia. APC, Annual Percentage Change (the annual percent change in the trend during each segment); AAPC, Average Annual Percentage Change (the weighted average of APCs over the entire study period); YLL, Years of Life Lost; NCDs, Non-communicable diseases.

Over the past 30 years, the global age-standardized YLL rate due to NCDs decreased −1.18/year (−1.2, −1.17), from 17353.86 (95%UI, 16593.95–17983.53) in 1990 to 11954.10 (95% UI, 11322.51–12588.20) in 2021. Among G20 countries, the reduction in YLL was 1.34%/year, with a 95% CI of −1.35 to −1.32%. Notably, China experienced a 2.08% (95% CI: −2.12 to −2.05) decrease in YLL due to NCDs. In the majority of G20 countries, the age-standardized of YLL associated with NCDs had declined. However, an exception to this trend was South Africa, which reported an increase in the YLL rate by 0.14%/year (95% CI: 0.04–0.23). The top five countries with the largest AAPC in the age-standardized YLL rate due to NCDs were the Republic of Korea, Australia, Italy, the United Kingdom, and France (Figure 3 and Table 1).

From 1990 to 2019, marked reductions in mortality rates attributed to level 2 causes, including CRDs, digestive diseases, cardiovascular diseases, other NCDs, and neoplasms were observed globally, in G20 countries, and in China (Table 3). However, despite the overall downward trend in NCD mortality for most level 2 causes, global mortality rates for neurological disorders, skin and subcutaneous diseases, and diabetes and kidney diseases exhibited persistent increases. The percentage changes in mortality for these conditions with 95% UI were 0.96% (−3.60 to 7.21), 6.46% (−4.97 to 20.20), and 14.79% (7.09–21.03), respectively. Among G20 countries, substantial mortality increases were identified for neurological disorders, substance use disorders, diabetes and kidney diseases, and mental disorders. Notably, mental disorders demonstrated the most pronounced escalation at 13.65% (95% UI: 4.28–23.95). A striking finding emerged from China, where mental disorder-related mortality surged by 514.01% (95% UI: 232.33–1658.05), representing an exceptionally rapid growth trajectory compared to global patterns. For deaths from level 3 causes, the largest increase was for interstitial lung disease and pulmonary sarcoidosis (50.31%, 95% UI: 31.72–74.28) in global, interstitial lung disease and pulmonary sarcoidosis (58.32, 95% UI: 39.26–82.01) in G20 countries, and other neurological disorders (546.52%, 95% UI: 320.94–854.48) in China.

In 2021, cardiovascular diseases constituted the predominant level 2 causes of YLL per 100,000 population globally (7,161.52, 95% UI: 6,806.73–7,430.69), among G20 nations (6,786.04, 95% UI: 6,429.08–7,073.83) and in China (7,698.41, 95% UI: 6,808.16–8,560.81) (Table 3 and Supplementary Figure 1). For level 3 causes, ischemic heart disease (2164.92, 95% UI: 2025.00–2283.85) were the leading causes of YLL in global and G20 countries (1985.97, 95% UI: 1836.50–2118.16) and stroke were the leading causes in China (2395.38, 95% UI: 2005.46–2828.64). Between 1990 and 2019, significant declines in YLL associated with level 2 causes—particularly CRDs, digestive diseases, and other NCDs were documented across global populations, G20 nations, and China. However, this overall downward trend in NCD-related YLL burden contrasted with notable exceptions. Diabetes and kidney diseases showed an 8.35% (95% UI: 0.53–14.88) increase in global YLL attributable to NCDs. Within G20 countries, three disease categories exhibited statistically significant YLL rate elevations: diabetes and kidney diseases (percentage change not specified), substance use disorders, and mental disorders. The latter demonstrated the most substantial increase at 13.52% (95% UI: 4.29–23.96). China presented a particularly remarkable epidemiological pattern, with mental disorder-associated YLL rates escalating by 504.15% (95% UI: 223.01–1655.07), a growth magnitude exceeding global trends by an order of magnitude. For YLL from level 3 causes, the largest increase was for other neurological disorders (37.26%, 95% UI: 18.22–52.87) in global, drug use disorders (44.00%, 95% UI:29.13–63.14) in G20 countries, and multiple myeloma (217.11%, 95% UI: 26.6–26.6]) in China.

### 3.3 Cross-country inequality analysis of NCDs from 1990 to 2021

**FIGURE 4:**
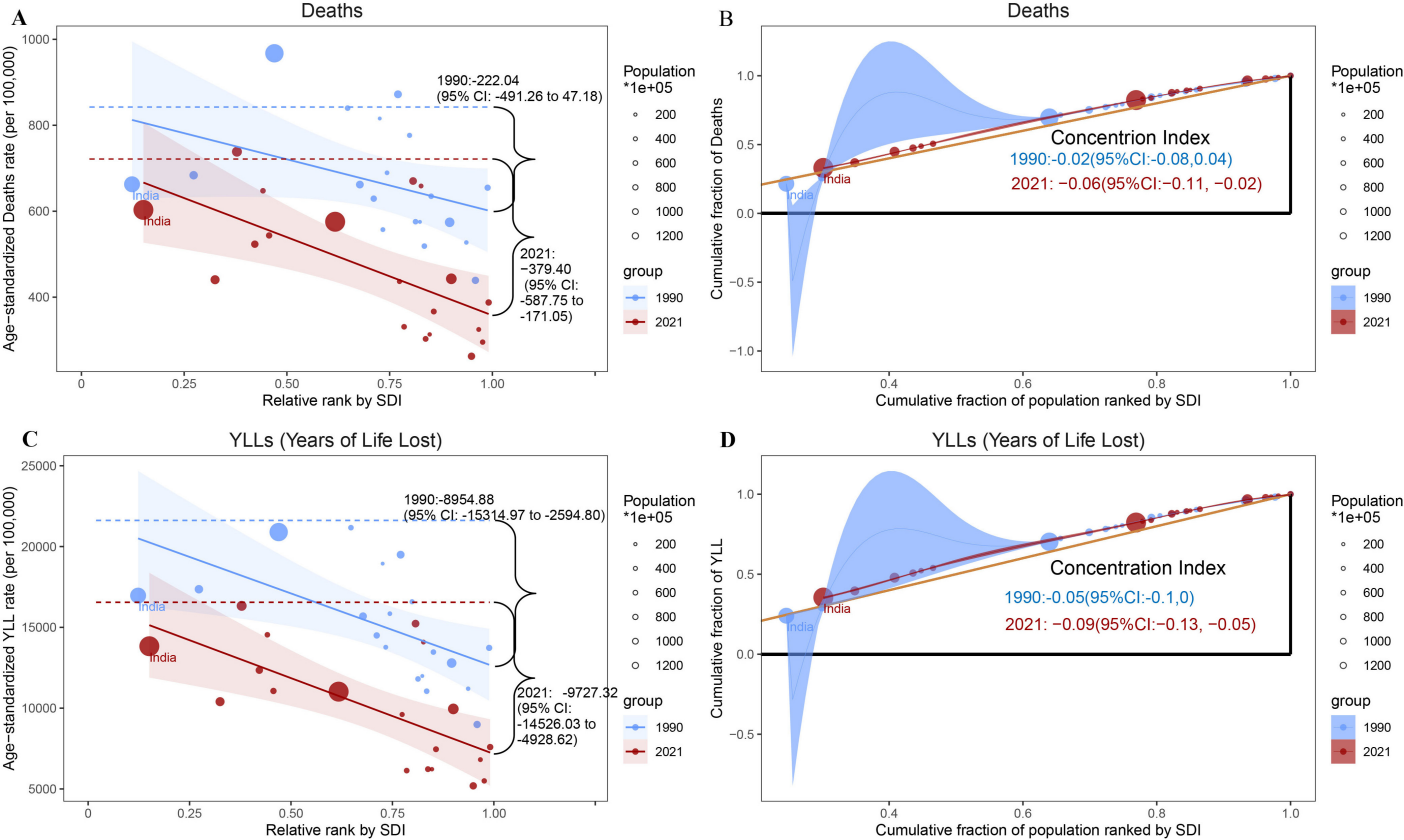
Absolute and relative cross-country inequality in ASMRs **(A,B)** and YLL rates **(C,D)** for NCDs among G20 countries. Panels A and C depict the SII for ASMR of NCDs. Scatter points represent country/regional data, with marker sizes proportional to population size. The fitted regression lines illustrate the strength of association between the SDI and disease burden. Panels **(B,D)** present the Concentration Index, which quantifies relative inequality through numerical integration of the area under the Lorenz curve. This curve maps the alignment between mortality and YLL distributions against the SDI-ranked population distribution. Data from 1990 and 2021 are denoted in blue and red, respectively. Colors: Blue = 1990; Red = 2021. SII, Slope Index of Inequality (absolute inequality). Concentration Index: A measure of relative inequality derived via numerical integration of the Lorenz curve. SDI, Socio-demographic Index; YLL, Years of Life Lost; ASMR, Age-standardized Mortality Rate.

From 1990 to 2021, the disease burden of NCDs in G20 countries showed significant and widening absolute and relative inequalities across the SDI gradient, with the NCD burden consistently higher in low-SDI countries than in high-SDI countries. SDI levels of G20 countries in 1990 and 2021 are presented in Supplementary Table 4. Negative values of the SII and Concentration Index indicate that the NCD burden was concentrated in countries with lower SDI. A larger negative magnitude of both indices directly reflects increasing absolute and relative inequality, respectively. The ASMRs and age-standardized YLL rates for NCDs in G20 countries showed significant increases in the magnitude of both absolute inequality (SII) and relative inequality (Concentration Index) (Figure 4). Specifically, in absolute inequality analyses, the SII for ASMRs increased from −222.04 (95% CI: −491.26 to 47.18) in 1990 to −379.40 (95% CI: −587.75 to −171.05) in 2021. Meanwhile, the SII for age-standardized YLL rates increased from −8954.88 (95% CI: −15314.97 to −2594.80) in 1990 to −9727.32 (95% CI: −14526.03 to −4928.62) in 2021. For relative inequality, the Concentration Index for ASMRs rose from −0.02 (95% CI: −0.08 to 0.04) in 1990 to −0.06 (95% CI: −0.11 to −0.02) in 2021. The Concentration Index for age-standardized YLL rates increased from −0.05 (95% CI: −0.10 to 0.00) in 1990 to −0.09 (95% CI: −0.13 to −0.05) in 2021. Gender-stratified analyses revealed similar trends in health inequalities in males and females. These results indicate that health inequalities in NCD burden across G20 countries have intensified over the past three decades along the SDI gradient. Both absolute and relative disparities between low- and high-SDI countries have widened. The NCD burden remains persistently concentrated in low-SDI nations, with similar patterns observed in both males and females.

### 3.4 Projected ASMR and age-standardized YLL rates trends in NCDs

**FIGURE 5:**
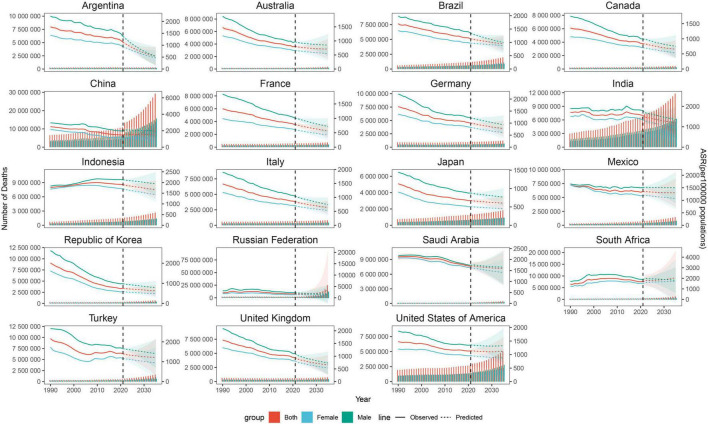
Temporal trend in the number of deaths for NCDs from 1990 to 2035 among G20 countries. Each panel represents a G20 country, showing the number of deaths (left *y*-axis, absolute count) and the age-standardized mortality rate (ASR) (right *y*-axis, per 100,000 population) from 1990 to 2035. Solid lines represent observed number of deaths, and dashed lines represent number of deaths predicted by the BAPC model. Red: Both sexes, Light blue: Female, Dark green: Male. NCDs, Non-communicable diseases; ASR, Age-standardized mortality rate (per 100,000 population); BAPC, Bayesian age-period-cohort model.

We employed the BAPC model to project the burden of NCDs from 2021 to 2035 (Figure 5, Supplementary Figure 2, and Supplementary Table 5). Between 2021 and 2035, ASMR and YLL rates demonstrated downward trends in most countries. However, China, the Russian Federation, and South Africa exhibited increasing ASMR trends, with projected rises from 764.69 (763.99–765.38), 669.87 (661.46–678.27), and 647.79 (628.70–666.89) in 2021 to 768.74 (443.49–1093.99), 1343.48 (0.00–8142.20), and 801.40 (72.19–1812.09) in 2035, respectively. Similarly, YLL rates in Saudi Arabia, South Africa, and the United States of America are projected to increase from 14,073.28 (13,921.38–14,225.18), 14,518.86 (14,427.29–14,610.43), and 9,940.72 (9,916.17–9,965.27) in 2021 to 15,682.54 (7,865.24–23,499.85), 16,781.02 (1,581.56–37,857.40), and 10,627.27 (5,461.67–15,792.86) in 2035. Gender-stratified projections revealed that in most countries, both males and females followed trends consistent with the overall population, though females exhibited more pronounced changes. However, in China, the ASMR of female is projected to decrease from 437.23 (436.82–437.64) in 2021 to 415.45 (120.93–709.96) in 2035, whereas the male ASMR is expected to rise from 574.44 (571.79–577.10) to 595.94 (248.47–943.42) during the same period. Contrasting gender patterns were observed in the Russian Federation and South Africa. By 2035, the burden of NCDs in most G20 nations is projected to decline substantially, with males demonstrating a marginally slower rate of reduction compared to females. Nevertheless, upward trends persist in certain countries, accompanied by varying degrees of sex-specific disparities.

## 4 Discussion

To our knowledge, this study provides the first systematic evaluation of NCDs burden across G20 nations from 1990 to 2021, employing YLL and mortality rates to quantify health disparities among economies representing 85% of global GDP. It not only provides important support for global research on the overall disease burden but also further highlights the core impact of such diseases on national health outcomes. Among Level 2 NCDs categories, cardiovascular diseases, neoplasms, and CRDs are the leading causes of death, and they also rank as the top three contributors to YLL. Although the age-standardized mortality rates and age-standardized YLL rates of most NCDs show a declining trend, significant variations remain across different countries and regions. This implies that the differentiation in disease burden among subnational regions deserves attention, and it is urgent to formulate health policies with both targeted and context-adaptive characteristics to address this issue.

Our results confirmed that NCDs have become the primary public health challenge in G20 countries. In 2021, NCDs accounted for 72.21% of the total mortality in G20 countries, which was significantly higher than the global average of 64.49%. This discrepancy reflects the population aging, lifestyle changes, and epidemiological transition experienced by G20 countries, with the disease burden shifting from infectious diseases to chronic conditions (Eufrasio Espinosa and Lenny Koh, 2024; Gong et al., 2024; Yang et al., 2025). Notably, the ASMR of NCDs in China was 1.14 times the average level of G20 countries, and NCDs accounted for 90.99% of China’s total mortality—indicating that NCDs have become the top threat to public health in China, which is consistent with the conclusions of previous domestic epidemiological studies (Liu et al., 2024; Peng et al., 2024).

A significant finding of this study is the substantial heterogeneity in NCD burden among G20 countries. Indonesia, the Russian Federation, and Saudi Arabia had the highest ASMRs, while Japan, the Republic of Korea, and France maintained the lowest levels. This gap stems from multiple factors: high-income countries such as Japan and the Republic of Korea have established mature universal healthcare systems, implemented comprehensive risk factor interventions (Nomura et al., 2022; Yoo et al., 2022); in contrast, low- and middle-income member states within the G20 face challenges including inadequate healthcare infrastructure, limited access to essential medicines, and the double burden of infectious diseases and NCDs (Kazibwe et al., 2021). In particular, Indonesia’s persistently high NCD burden is closely associated with its high smoking prevalence, unhealthy dietary patterns, insufficient access to primary care, and relatively weak chronic disease management systems (Lisetyaningrum et al., 2021; Marthias et al., 2021; GBD 2021 Tobacco Forecasting Collaborators, 2024). Meanwhile, Russia exhibits a high NCD mortality trend largely driven by widespread tobacco use, heavy alcohol consumption, regional inequities in healthcare resource allocation, and relatively limited access to standardized chronic disease care. The high and sustained NCD burden in Saudi Arabia may be partially linked to rapid population aging, physical inactivity, and metabolic risk factors including obesity and diabetes (Boettiger et al., 2023).

From 1990 to 2021, health inequalities related to NCDs among G20 countries exhibited a systemic escalation. Consistent with our findings on inequality indices, this escalation is reflected by the increasingly negative magnitudes of the Slope Index of Inequality (SII) and Concentration Index, which directly indicate a widening of absolute and relative disparities in NCD burden across the SDI gradient. Disparities in ASMR and YLL gradually emerged between regions with low and high SDI, with disease burdens disproportionately concentrated in socioeconomically vulnerable populations. This phenomenon reveals a negative correlation between socioeconomic development and health burden, which may stem from the scarcity of medical resources, lagging chronic disease management, and uneven distribution of social determinants of health in low-SDI regions (Ghiasi and Weech-Maldonado, 2024). Specifically, high-income economies and developing economies have demonstrated distinctly different disease transition trajectories. Japan maintains a world-leading low NCD mortality rate, while Indonesia faces an extremely high disease burden. This discrepancy highlights the implementation gaps of cost-effective interventions recommended by the WHO across countries, a finding also confirmed by Armocida et al. (2022) who reported substantial cross-national variations in NCD burden. Notably, although 78% of G20 member states have experienced declining mortality rates, South Africa has seen a paradoxical surge of 16.23% in NCD mortality, a phenomenon correlated with the scaling-up of antiretroviral therapy and reflecting the complex characteristics of HIV-NCD comorbidity. In addition, South Africa’s fragmented healthcare system, uneven access to chronic disease management, and relatively high prevalence of multiple behavioral and cardiometabolic risk factors further impede the decline of NCD outcomes. Essentially, these regional disparities reflect systemic inequalities in regional economic development and healthcare infrastructure. Therefore, to targetedly reduce NCD mortality, G20 countries—particularly high-burden nations such as Indonesia, Russia, Saudi Arabia, and South Africa—urgently need to implement WHO-recommended interventions and simultaneously strengthen healthcare system development.

Cardiovascular diseases remained the leading cause of mortality globally and among G20 countries in 2021, and despite a nearly 40% reduction in their ASMR and YLL rate since 1990, they continue to be the primary chronic disease threatening the health of G20 residents. The continuous increase in the burden of cardiovascular diseases may be mainly attributed to atherosclerotic diseases, with high blood pressure, dietary risks, and high cholesterol as the core driving factors (Chong et al., 2025). Due to the change in lifestyle and eating habits, the accumulation of risk factors in childhood is an increasing problem (Wolska et al., 2025). Among modifiable cardiovascular risk factors, metabolic diseases (including hypertension, low-density lipoprotein hypercholesterolemia, diabetes mellitus, and obesity) play a crucial role (Olié et al., 2024). The formulation of national-level relevant policies may facilitate early detection and improvements in acute management, thereby reducing the disease burden. Such as the guidelines for the management of patients with acute coronary syndrome issued by the American College of Cardiology/American Heart Association (Rao et al., 2025), the management strategies for chronic inflammatory diseases and cardiovascular risk (Cacciatore et al., 2025). Among Level 3 causes, ischemic heart disease and stroke have consistently been ranked as the top two contributors to NCD-related deaths since 1990. In 2021, the ASMR and YLL rate of ischemic heart disease and stroke decreased significantly, yet its burden remains substantial. This suggests that stroke and ischemic heart disease should be prioritized in the prevention and control strategies for cardiovascular diseases among G20 countries.

In 2021, neoplasm was the second leading cause of NCD-related deaths and YLL among G20 countries. Compared with 1990, the ASMR and YLL incidence of cancer have dropped by more than 20%. Nevertheless, the overall burden remains heavy. Over the past few decades, the world and G20 countries have built a systematic approach for cancer prevention and control featuring global framework leadership, country-specific differentiated actions, and multi-dimensional collaborative advancement. At the global level, with the WHO Global Strategy for Cancer Prevention and Control, NCD Action Plans (Hategeka et al., 2022; Lv and Zhang, 2024), and the 90–70–90 targets for cervical cancer elimination as the core (Zhao et al., 2024), primary prevention measures such as tobacco and alcohol control and prevention of infectious etiologies have been implemented (Gottlieb et al., 2024). G20 countries have developed differentiated practices aligned with their own development levels. High-income member states (including the United States, European countries, Japan, and Australia) have leveraged national strategies—such as the U.S. Cancer Moonshot (Singer, 2022) and the European Union’s Beating Cancer Plan (Delnord et al., 2024)—to focus on precision screening [e.g., gastric cancer screening in Japan (Narii et al., 2022) and skin cancer prevention and control in Germany (Garbe et al., 2023)] and innovations in targeted/immunotherapy. Emerging member states (including China, India, and Brazil) have prioritized basic prevention and control measures, advancing tobacco control, HPV vaccination, and the construction of grassroots screening networks (Nascimento et al., 2021; Burki, 2023; Chan et al., 2023).

CRDs ranked as the third leading cause of NCD-related deaths in G20 countries in 2021. Since 1990, the ASMR and YLL rate due to CRDs have decreased by over 35%. Among the three-level etiological factors associated with CRDs, chronic obstructive pulmonary disease (COPD) remains the primary contributor—its ASMR and YLL rate have dropped by approximately 40% since 1990. This shift in epidemiological characteristics is the result of the synergistic effects of multi-dimensional prevention and control strategies implemented by G20 countries, while also reflecting the existing challenges in disease prevention and control. The declining trend is primarily attributed to tobacco control measures adopted by G20 countries (Lu et al., 2024). Additionally, the upgrading of air quality standards, the control of industrial and traffic pollution, and the promotion of clean energy have also reduced the risk of population-wide lung function impairment by minimizing environmental triggers (Weng et al., 2023). Furthermore, the improvement of healthcare systems in G20 countries has further driven down the disease burden: the popularization of pulmonary function testing at the primary healthcare level has enabled early screening and diagnosis of COPD and the reform of rehabilitation therapy and medical security systems has improved patients’ treatment compliance and quality of life (Shibuya et al., 2022; Adams et al., 2023). In the future, G20 countries should strengthen international cooperation to promote the sharing of technologies and policy experiences in tobacco control and pollution management, and enhance the global accessibility of diagnostic and therapeutic drugs for respiratory diseases.

NCDs exhibited pronounced gender and age specific mortality patterns globally and within G20 countries. Males consistently demonstrated higher ASMR and YLL than females. Potentially attributable to biological vulnerabilities and behavioral risks (Wilkinson et al., 2022; Harvey and Alvarez de la Rosa, 2025). Testosterone levels in males may increase infection risk by suppressing immune responses, while estrogen exhibits immune-modulating properties that mitigate autoimmune disease susceptibility in females (Lakshmikanth et al., 2024). A bimodal age distribution emerged, with mortality peaks among children under five and individuals aged ≥ 95 years. Prolonged Th2 immune dominance in children under five, coupled with markedly reduced antiviral interferon secretion capacity (31% of adult levels), predisposes this population to elevated risks of chronic progression post-infection (Villavicencio et al., 2024). Exposure to a deteriorating environment during pregnancy may concurrently harm fetal development and accelerate cellular senescence through dual mechanisms of teratogenic stress and oxidative damage (Scaife et al., 2021). Notably, China achieved exceptional progress in pediatric health outcomes. Analysis of gender differences shows that the improvement of men’s health is generally lagging behind. In China, a unique phenomenon has emerged where women’s ASMR has declined while men’s has risen against the trend.

According to our projections, the burden of NCDs is expected to decline in most countries by 2035, which underscores the effectiveness of global NCDs prevention and control strategies. Nevertheless, the ASMR and YLL are projected to remain on an upward trajectory in China, Russia, and South Africa. This divergent trend can be attributed to a combination of multifaceted determinants. From a socio-environmental perspective, Russia is characterized by high tobacco prevalence (Merkin et al., 2021), and unequal regional distribution of healthcare resources, both of which exacerbate NCD-related risks. In South Africa, the heavy burden of HIV/AIDS–NCD comorbidity, together with a fragmented healthcare system, has further amplified adverse health outcomes (Gizamba et al., 2023). Meanwhile, China is undergoing a critical demographic and epidemiological transition featured by rapid population aging and substantial shifts in lifestyle patterns (The Lancet, 2022). Environmental challenges associated with industrialization and urbanization, coupled with suboptimal adherence to chronic disease management in certain populations, have collectively impeded the simultaneous reduction of NCD burden compared with other countries (Liang et al., 2024). Gender disparities and aging populations remain critical gaps in global NCD governance, requiring life-course approaches and gender-specific interventions to reduce mortality burdens. However, there are also some limitations of this study. First, the GBD database mainly uses existing reports and studies rather than direct country data, which may affect the timeliness and quality of information—especially in poorer areas. While GBD uses advanced methods to adjust data, these issues can still impact accuracy. Second, differences in how countries track and report diseases (like diagnosis and record-keeping) make it hard to compare data fairly. Such gaps often lead to missing cases, and the real disease burden might only be seen through detailed local tracking in high-risk areas.

## 5 Conclusion

In summary, this study utilized data from GBD 2021 to delineate the burden and temporal trends of NCDs across G20 countries from 1990 to 2021 and prediction to 2035. While overall NCD mortality has declined, concerning increases persist in specific regions, with marked disparities across gender and age groups—particularly higher burdens among males and a bimodal age distribution. Cardiovascular diseases remain the leading cause of death, while neurological and mental health disorders emerge as rapidly growing threats. The findings underscore the need for prioritized interventions targeting high-risk populations, equitable resource allocation, and integrated health strategies to address the complex epidemiological transition in an area of demographic and environmental change.

## Data Availability

The original contributions presented in the study are included in the article/Supplementary material, further inquiries can be directed to the corresponding author.

## References

[B1] Abdulrahim A. O. Doddapaneni N. S. P. Salman N. Giridharan A. Thomas J. Sharma K. et al. (2025). The gut-heart axis: A review of gut microbiota, dysbiosis, and cardiovascular disease development. *Ann. Med. Surg.* 87 177–191. 10.1097/ms9.0000000000002789 40109640 PMC11918638

[B2] Adams S. J. Stone E. Baldwin D. R. Vliegenthart R. Lee P. Fintelmann F. J. (2023). Lung cancer screening. *Lancet* 401 390–408. 10.1016/s0140-6736(22)01694-4 36563698

[B3] Armocida B. Monasta L. Sawyer S. Bustreo F. Segafredo G. Castelpietra G. et al. (2022). Burden of non-communicable diseases among adolescents aged 10-24 years in the EU, 1990-2019: A systematic analysis of the Global burden of diseases study 2019. *Lancet Child Adolesc. Health* 6 367–383. 10.1016/s2352-4642(22)00073-6 35339209 PMC9090900

[B4] Barati H. Pourhoseingholi M. A. Roshandel G. Nazari S. S. H. (2024). Bayesian age-period-cohort projection of cancers in Iran: A modeling study. *BMC Cancer* 24:1538. 10.1186/s12885-024-13289-0 39696020 PMC11657364

[B5] Boettiger D. C. Lin T. K. Almansour M. Hamza M. M. Alsukait R. Herbst C. H. et al. (2023). Projected impact of population aging on non-communicable disease burden and costs in the Kingdom of Saudi Arabia, 2020-2030. *BMC Health Serv. Res.* 23:1381. 10.1186/s12913-023-10309-w 38066590 PMC10709902

[B6] Burki T. K. (2023). India rolls out HPV vaccination. *Lancet Oncol.* 24:e147. 10.1016/s1470-2045(23)00118-3 36934729

[B7] Cacciatore S. Andaloro S. Bernardi M. Oterino Manzanas A. Spadafora L. Figliozzi S. et al. (2025). Chronic inflammatory diseases and cardiovascular risk: Current insights and future strategies for optimal management. *Int. J. Mol. Sci.* 26:3071. 10.3390/ijms26073071 40243756 PMC11989023

[B8] Cao F. He Y. S. Wang Y. Zha C. K. Lu J. M. Tao L. M. et al. (2023). Global burden and cross-country inequalities in autoimmune diseases from 1990 to 2019. *Autoimmun. Rev.* 22:103326. 10.1016/j.autrev.2023.103326 36958621

[B9] Chan K. H. Xiao D. Zhou M. Peto R. Chen Z. (2023). Tobacco control in China. *Lancet Public Health* 8 e1006–e1015. 10.1016/s2468-2667(23)00242-6 38000880

[B10] Chong B. Jayabaskaran J. Jauhari S. M. Chan S. P. Goh R. Kueh M. T. W. et al. (2025). Global burden of cardiovascular diseases: Projections from 2025 to 2050. *Eur. J. Prev. Cardiol.* 32 1001–1015. 10.1093/eurjpc/zwae281 39270739

[B11] Delnord M. Schittecatte G. Ghattas J. Van Den Bulcke M. (2024). Introduction to the Supplement ‘Coming together to fight cancer: A series of policy briefs taking stock of the implementation of Europe’s beating cancer plan in Belgium’. *Arch. Public Health* 82:164. 10.1186/s13690-024-01383-5 39327629 PMC11426068

[B12] Eufrasio Espinosa R. M. Lenny Koh S. C. (2024). Forecasting the ecological footprint of G20 countries in the next 30 years. *Sci. Rep.* 14:8298. 10.1038/s41598-024-57994-z 38594329 PMC11004171

[B13] FDI World Dental Federation. (2025). Oral health and noncommunicable diseases (n.d.). *Int. Dent. J.* 75 11–12. 10.1016/j.identj.2024.12.008 39842948 PMC11806323

[B14] Garbe C. Augustin M. Augustin J. Baltus H. Eisemann N. Hübner J. et al. (2023). Evaluation of skin cancer screening in Germany − participation, tumor detection and interval tumors based on SHI data. *J. Dtsch. Dermatol. Ges.* 21 3–11. 10.1111/ddg.15170 38063281

[B15] GBD 2021 Diabetes Collaborators. (2023). Global, regional, and national burden of diabetes from 1990 to 2021, with projections of prevalence to 2050: A systematic analysis for the Global burden of disease study 2021. *Lancet* 402 203–234. 10.1016/s0140-6736(23)01301-6 37356446 PMC10364581

[B16] GBD 2021 Tobacco Forecasting Collaborators (2024). Forecasting the effects of smoking prevalence scenarios on years of life lost and life expectancy from 2022 to 2050: A systematic analysis for the Global burden of disease study 2021. *Lancet Public Health* 9 e729–e744. 10.1016/s2468-2667(24)00166-x 39366729 PMC11447278

[B17] Ghiasi A. Weech-Maldonado R. (2024). The Moderating effect of the social deprivation index (SDI) on the Relationship between hospital strategy and financial performance. *Hosp. Top.* 102 173–183. 10.1080/00185868.2022.2114965 36000721

[B18] Gizamba J. M. Davies J. Africa C. Choo-Kang C. Goedecke J. H. Madlala H. et al. (2023). Prevalence of obesity, hypertension and diabetes among people living with HIV in South Africa: A systematic review and meta-analysis. *BMC Infect. Dis.* 23:861. 10.1186/s12879-023-08736-5 38062372 PMC10704741

[B19] Gong Y. Jiang Q. Zhai M. Tang T. Liu S. (2024). Thyroid cancer trends in China and its comparative analysis with G20 countries: Projections for 2020-2040. *J. Glob. Health* 14:04131. 10.7189/jogh.14.04131 38873786 PMC11177899

[B20] Gottlieb S. L. Spielman E. Abu-Raddad L. Aderoba A. K. Bachmann L. H. Blondeel K. et al. (2024). WHO global research priorities for sexually transmitted infections. *Lancet Glob. Health* 12 e1544–e1551. 10.1016/s2214-109x(24)00266-3 39043199 PMC11342064

[B21] Harvey B. J. Alvarez de la Rosa D. (2025). Sex differences in kidney health and disease. *Nephron* 149 77–103. 10.1159/000541352 39406203

[B22] Hategeka C. Adu P. Desloge A. Marten R. Shao R. Tian M. et al. (2022). Implementation research on noncommunicable disease prevention and control interventions in low- and middle-income countries: A systematic review. *PLoS Med.* 19:e1004055. 10.1371/journal.pmed.1004055 35877677 PMC9359585

[B23] Kazibwe J. Tran P. B. Annerstedt K. S. (2021). The household financial burden of non-communicable diseases in low- and middle-income countries: A systematic review. *Health Res. Policy Syst.* 19:96. 10.1186/s12961-021-00732-y 34154609 PMC8215836

[B24] Kim H. J. Fay M. P. Feuer E. J. Midthune D. N. (2000). Permutation tests for joinpoint regression with applications to cancer rates. *Stat. Med.* 19 335–351. 10.1002/(sici)1097-0258(20000215)19:3<335::aid-sim336<3.0.co;2-z 10649300

[B25] Lakshmikanth T. Consiglio C. Sardh F. Forlin R. Wang J. Tan Z. et al. (2024). Immune system adaptation during gender-affirming testosterone treatment. *Nature* 633 155–164. 10.1038/s41586-024-07789-z 39232147 PMC11374716

[B26] Li K. J. Burton-Pimentel K. J. Vergères G. Feskens E. J. M. Brouwer-Brolsma E. M. (2022). Fermented foods and cardiometabolic health: Definitions, current evidence, and future perspectives. *Front. Nutr.* 9:976020. 10.3389/fnut.2022.976020 36204374 PMC9530890

[B27] Li S. X. Guo Y. (2023). Gut microbiome: New perspectives for type 2 diabetes prevention and treatment. *World J. Clin. Cases* 11 7508–7520. 10.12998/wjcc.v11.i31.7508 38078135 PMC10698456

[B28] Liang S. Chen Y. Sun X. Dong X. He G. Pu Y. et al. (2024). Long-term exposure to ambient ozone and cardiovascular diseases: Evidence from two national cohort studies in China. *J. Adv. Res.* 62 165–173. 10.1016/j.jare.2023.08.010 37625570 PMC11331174

[B29] Lisetyaningrum I. Pujasari H. Kuntarti K. (2021). A cross-sectional analysis of snacking habits, eating habits, physical activity, and indicators of obesity among high school students in Jakarta, Indonesia. *J. Public Health Res.* 10:2404. 10.4081/jphr.2021.2402 34060755 PMC9309651

[B30] Liu H. Yin P. Qi J. Zhou M. (2024). Burden of non-communicable diseases in China and its provinces, 1990-2021: Results from the global burden of disease study 2021. *Chin. Med. J.* 137 2325–2333. 10.1097/cm9.0000000000003270 39193717 PMC11441934

[B31] Lu W. Aarsand R. Schotte K. Han J. Lebedeva E. Tsoy E. et al. (2024). Tobacco and COPD: Presenting the World health organization (WHO) tobacco knowledge summary. *Respir. Res.* 25:338. 10.1186/s12931-024-02961-5 39261873 PMC11391604

[B32] Lv J. Zhang Z. F. (2024). Progress and challenges in NCD prevention and control in China. *Bmj* 387:q2098. 10.1136/bmj.q2098 39424318 PMC11484825

[B33] Ma H. Mu X. Jin Y. Luo Y. Wu M. Han Z. (2024). Multimorbidity, lifestyle, and cognitive function: A cross-cultural study on the role of diabetes, cardiovascular disease, cancer, and chronic respiratory diseases. *J. Affect. Disord.* 362 560–568. 10.1016/j.jad.2024.07.053 39019233

[B34] Manzanera M. (2025). From microbial consortia to ecosystem resilience: The integrative roles of holobionts in stress biology. *Biology* 14:1203. 10.3390/biology14091203 41007348 PMC12466951

[B35] Marthias T. Anindya K. Ng N. McPake B. Atun R. Arfyanto H. et al. (2021). Impact of non-communicable disease multimorbidity on health service use, catastrophic health expenditure and productivity loss in Indonesia: A population-based panel data analysis study. *BMJ Open* 11:e041870. 10.1136/bmjopen-2020-041870 33597135 PMC7893673

[B36] Martinez R. Lloyd-Sherlock P. Soliz P. Ebrahim S. Vega E. Ordunez P. et al. (2020). Trends in premature avertable mortality from non-communicable diseases for 195 countries and territories, 1990-2017: A population-based study. *Lancet Glob. Health* 8 e511–e523. 10.1016/s2214-109x(20)30035-8 32199120

[B37] Martins D. Silva C. Ferreira A. C. Dourado S. Albuquerque A. Saraiva F. et al. (2024). Unravelling the gut microbiome role in cardiovascular disease: A systematic review and a meta-analysis. *Biomolecules* 14:731. 10.3390/biom14060731. 38927134 PMC11201797

[B38] Merkin A. Nikolaev A. Nikoforov I. Komarov A. Glover M. (2021). Trends in tobacco smoking and smoking cessation in Russia with a focus on Indigenous populations: A narrative review. *Glob. Epidemiol.* 3:100043. 10.1016/j.gloepi.2020.100043 37635728 PMC10446109

[B39] Narii N. Sobue T. Zha L. Kitamura T. Iwasaki M. Inoue M. et al. (2022). Effectiveness of endoscopic screening for gastric cancer: The Japan public health center-based prospective study. *Cancer Sci.* 113 3922–3931. 10.1111/cas.15545 36002149 PMC9633299

[B40] Nascimento J. H. F. Vieira A. T. S. Souza Filho B. M. Tomaz S. C. Delgado Bocanegra R. E. Melo Costa V. S. et al. (2021). Breast cancer in Brazil: Screening program and surgical approach. *Cancer Epidemiol.* 73:101970. 10.1016/j.canep.2021.101970 34216956

[B41] NCD Countdown 2030 Collaborators (2020). NCD countdown 2030: Pathways to achieving sustainable development goal target 3.4. *Lancet* 396 918–934. 10.1016/s0140-6736(20)31761-x 32891217 PMC7470795

[B42] Nomura S. Sakamoto H. Ghaznavi C. Inoue M. (2022). Toward a third term of Health Japan 21 − implications from the rise in non-communicable disease burden and highly preventable risk factors. *Lancet Reg. Health West Pac.* 21:100377. 10.1016/j.lanwpc.2021.100377 35098183 PMC8783949

[B43] Olié V. Gabet A. Grave C. Helft G. Fosse-Edorh S. Piffaretti C. et al. (2024). Epidemiology of cardiovascular risk factors: Non-behavioural risk factors. *Arch. Cardiovasc. Dis.* 117 761–769. 10.1016/j.acvd.2024.08.005 39516129

[B44] Peng W. Chen S. Chen X. Ma Y. Wang T. Sun X. et al. (2024). Trends in major non-communicable diseases and related risk factors in China 2002-2019: An analysis of nationally representative survey data. *Lancet Reg. Health West Pac.* 43:100809. 10.1016/j.lanwpc.2023.100809 38456095 PMC10920046

[B45] Rao S. V. O’Donoghue M. L. Ruel M. Rab T. Tamis-Holland J. E. Alexander J. H. et al. (2025). 2025 ACC/AHA/ACEP/NAEMSP/SCAI guideline for the management of patients with acute coronary syndromes: A report of the American college of cardiology/American heart association joint committee on clinical practice guidelines. *Circulation* 151 e771–e862. 10.1161/cir.0000000000001309 40014670

[B46] Scaife P. J. Simpson A. Kurlak L. O. Briggs L. V. Gardner D. S. Broughton Pipkin F. et al. (2021). Increased placental cell senescence and oxidative stress in women with pre-eclampsia and normotensive post-term pregnancies. *Int. J. Mol. Sci.* 22:7295. 10.3390/ijms22147295 34298913 PMC8303298

[B47] Shah H. Ng T. L. (2023). A narrative review from gut to lungs: Non-small cell lung cancer and the gastrointestinal microbiome. *Transl. Lung Cancer Res.* 12 909–926. 10.21037/tlcr-22-595 37197624 PMC10183407

[B48] Shibuya M. Yamamoto S. Kobayashi S. Nishie K. Yamaga T. Kawachi S. et al. (2022). Pulmonary rehabilitation for patients after COPD exacerbation. *Respir. Care* 67 360–369. 10.4187/respcare.09066 34876493

[B49] Singer D. S. (2022). A new phase of the cancer moonshot to end cancer as we know it. *Nat. Med.* 28 1345–1347. 10.1038/s41591-022-01881-5 35760861 PMC9244436

[B50] The Lancet (2022). Population ageing in China: Crisis or opportunity? *Lancet* 400:1821. 10.1016/s0140-6736(22)02410-2 36436518

[B51] Villavicencio F. Perin J. Eilerts-Spinelli H. Yeung D. Prieto-Merino D. Hug L. et al. (2024). Global, regional, and national causes of death in children and adolescents younger than 20 years: An open data portal with estimates for 2000-21. *Lancet Glob. Health* 12 e16–e17. 10.1016/s2214-109x(23)00496-5 37898143

[B52] Wang H. Song Y. Ma J. Ma S. Shen L. Huang Y. et al. (2023). Burden of non-communicable diseases among adolescents and young adults aged 10-24 years in the South-East Asia and Western Pacific regions, 1990-2019: A systematic analysis for the Global burden of disease study 2019. *Lancet Child Adolesc. Health* 7 621–635. 10.1016/s2352-4642(23)00148-7 37524095

[B53] Weng Z. Tong D. Wu S. Xie Y. (2023). Improved air quality from China’s clean air actions alleviates health expenditure inequality. *Environ. Int.* 173:107831. 10.1016/j.envint.2023.107831 36805809

[B54] WHO. (2024). *Noncommunicable Diseases.* Geneva: WHO.

[B55] Wilkinson N. M. Chen H. C. Lechner M. G. Su M. A. (2022). Sex differences in immunity. *Annu. Rev. Immunol.* 40 75–94. 10.1146/annurev-immunol-101320-125133 34985929 PMC9805670

[B56] Wolska M. Peruzzi M. Kaziród-Wolski K. Wróbel P. Oleś I. Sielski J. et al. (2025). Risk factors for cardiovascular diseases: The focus on primary prevention. *Minerva Cardiol. Angiol.* 73 245–253. 10.23736/s2724-5683.23.06360-3 37971709

[B57] Yang P. Huang W. Xu Y. Teng Y. Shu P. (2025). Trends and projections of the burden of gastric cancer in China and G20 countries: A comparative study based on the global burden of disease database 2021. *Int. J. Surg.* 111 4854–4865. 10.1097/js9.0000000000002464 40359560

[B58] Yoo S. H. Jung S. H. Shin S. J. (2022). Evaluation of an oral health management project in connection to a non-communicable disease prevention and management project: A case study in South Korea. *Int. J. Environ. Res. Public Health* 19:5209. 10.3390/ijerph19095209 35564607 PMC9102545

[B59] Yuan L. Tao J. Wang J. She W. Zou Y. Li R. et al. (2025). Global, regional, national burden of asthma from 1990 to 2021, with projections of incidence to 2050: A systematic analysis of the global burden of disease study 2021. *EClinicalMedicine* 80:103051. 10.1016/j.eclinm.2024.103051 39867965 PMC11764843

[B60] Zarocostas J. (2024). Brazil-led G20 to target hunger, poverty, and inequities. *Lancet* 404:1914. 10.1016/s0140-6736(24)02516-9 39551054

[B61] Zhang T. Jiang H. Xu X. Zhao Z. Zhou M. (2026). Non-communicable disease burden in China, 1990-2023: Evidence from the global burden of disease study 2023. *Chin. Med. J.* 139 48–57. 10.1097/cm9.0000000000003898 41199464 PMC12767929

[B62] Zhao F. Lang J. Qiao Y. Zhu L. (2024). How can China achieve WHO’s 2030 targets for eliminating cervical cancer? *Bmj* 386:e078641. 10.1136/bmj-2023-078641 39214542 PMC11359838

