# Supplementary material for: Burden and temporal trends of non-communicable diseases from 1990 to 2021 and prediction to 2035 in the group of twenty countries: a systematic analysis of the Global Burden of Disease Study 2021
Source: Front Microbiol. 2026 May 19;17:1851514. doi: 10.3389/fmicb.2026.1851514 (PMC13226602; doi:10.3389/fmicb.2026.1851514)
Supplement: Supplementary file 1 [file Data_Sheet_1.docx]

Supplementary Material

**1 Supplementary Figures**


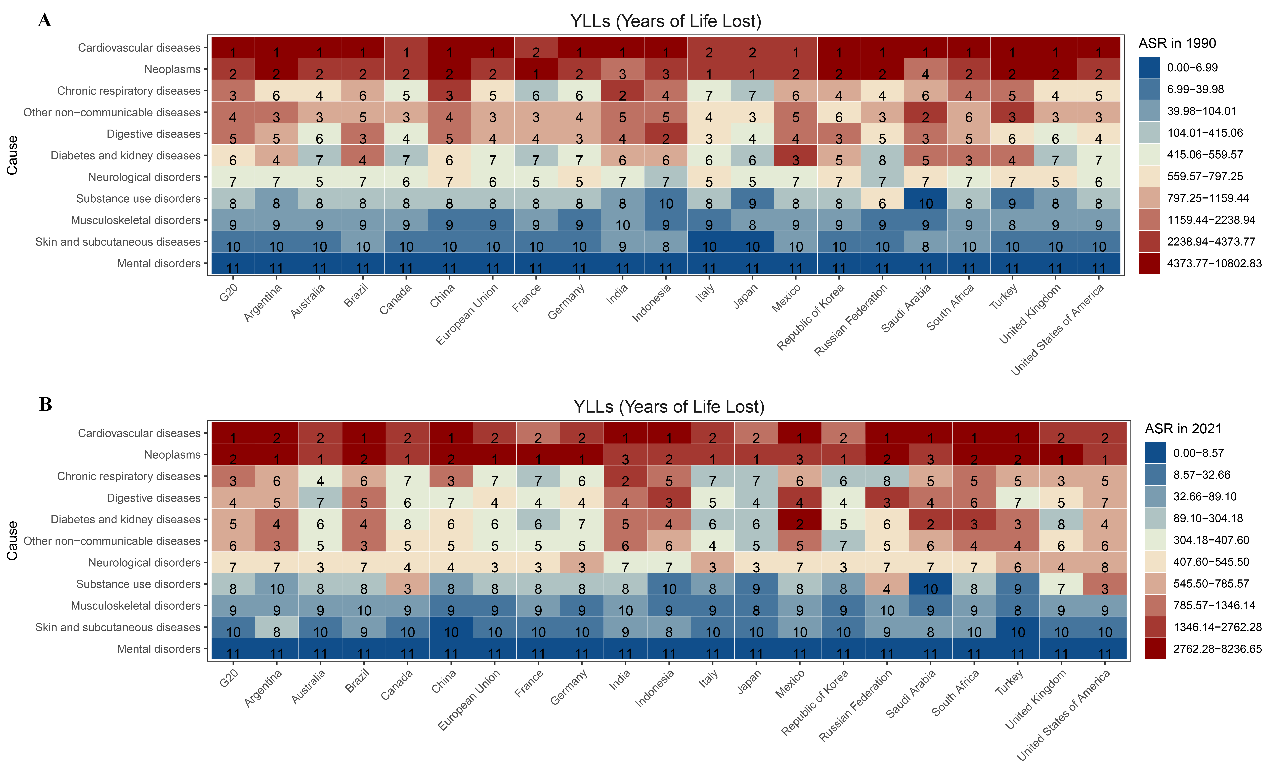


**Supplementary Figure 1**. **Ranking of YLL for NCDs across geographic regions in 1990 (A) and 2021 (B).** The colors in the figure represent rankings from high (red) to low (blue), A chromatic gradient scale (red: highest; blue: lowest) visualizes mortality rankings, with numerical annotations denoting the ordinal positions of 11 types of NCDs in G20 countries.


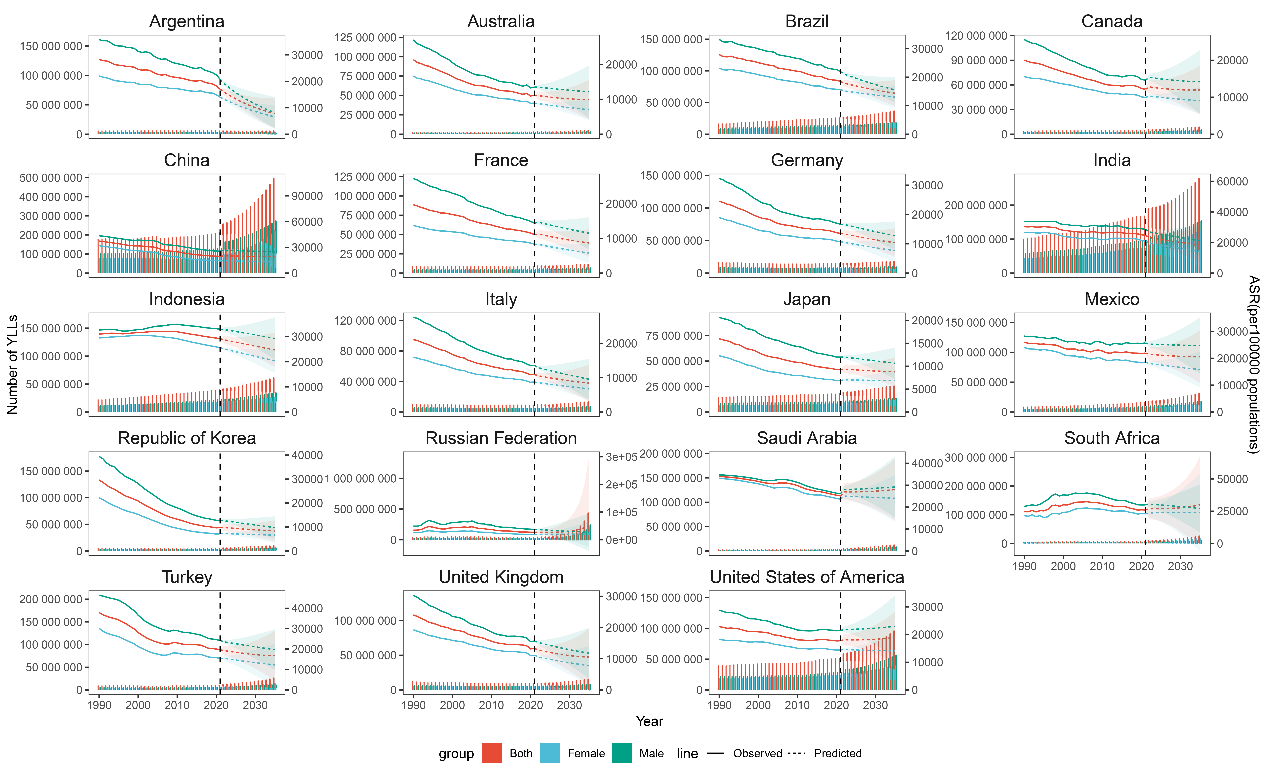


**Supplementary Figure 2. Temporal trend in the number of YLL for NCDs from 1990 to 2035 among G20 countries.** Solid lines represent observed number of deaths, and dashed lines represent number of deaths predicted by the BAPC model.

**2 Supplementary Tables**

| Supplementary Table 1 Prediction Code of the BAPC Model for Forecasting NCDs Burden in G20 Countries |
| --- |
| library(easyGBDR)  library(ggplot2)  df <- GBDread("IHME-GBD_2021_DATA1.zip")  bapc_results <- GBDbapc_prediction(  data = df,  measure_name =unique(df$measure),  cause_name =unique(df$cause),  location_name =unique(df$location),  rei_name = NULL,  predyear = 2035,  full_age_adjusted = F,  rate_lessen = NULL,  pop_predict = "GBD")  ggprediction_Dx(  data = bapc_results,  ratio = "auto",  CI = F,  predict_start = 2022,  group_name = "sex",  location_name = c("G20", "Argentina" ,"Australia" ,  "Brazil","Canada","China" ,"European Union",  "France","Germany","India" ,"Indonesia","Italy" ,"Japan",  "Mexico","Republic of Korea","Russian Federation",  "Saudi Arabia","South Africa","Turkey", "United Kingdom",  "United States of America"),  measure_name = "YLL","Death",  cause_name = unique(df$cause),  rei_name = NULL,) +  scale_fill_simpsons()+  scale_color_jco()+  facet_wrap(~location,scales = "free_y",ncol=4)+  theme_minimal()+  theme(legend.position = "bottom",  strip.text = element_text(size = 12),  strip.background = element_blank(), # 隐藏分面背景  axis.title = element_text(face = "bold", size = 14,margin = margin(t = 10, b = 10)),  # 坐标轴刻度标签加粗  axis.text = element_text(face = "bold", size = 12),  axis.text.x = element_text(  angle = 45, # 倾斜角度（45度）  hjust = 1, # 水平对齐方式（1=右对齐，避免标签超出绘图区）  vjust = 1 # 垂直对齐方式（可选，微调垂直位置）  ),  axis.line=element_line(linewidth = 0.8),  axis.ticks = element_line(size = 0.6),  panel.background = element_rect(fill = "white", color = NA),  plot.background = element_rect(fill = "white", color = NA),  panel.grid.major = element_blank(),  panel.grid.minor = element_blank()  ) |

**Supplementary Table 2** Number of NCDs deaths and YLL in Global and G20 countries, 1990-2021

| Countries | Deaths | | | YLL | | |
| --- | --- | --- | --- | --- | --- | --- |
|  | 1990 | 2021 | Absolute change,  1990-2021 | 1990 | 2021 | Absolute change,  1990-2021 |
| Global | 26775430.23(25836274.70-27603851.67) | 43768181.66(41841301.44-45882469.41) | 0.63%  (0.55 to 0.73) | 744258853.10(704672352.24-776045606.22) | 995388290.48(943244092.17-1046186372.57) | 0.34%  (0.25 to 0.44) |
| G20 | 20068960.54(19333304.65-20772956.97) | 32177966.39(30556993.40-34032507.93) | 0.60%  (0.50 to 0.72) | 523581369.25(498985804.71-545166016.87) | 676513247.05(640749099.39-719276662.21) | 0.29%  (0.21 to 0.40) |
| Argentina | 208924.72(207814.63-210323.43) | 247623.44(241767.31-253404.86) | 0.19%  (0.16 to 0.21) | 5056560.42(5021404.47-5097091.32) | 5081740.31(4951566.41-5210563.10) | 0.00%  (-0.02 to 0.03) |
| Australia | 107967.09(107325.95-108634.85) | 154738.68(153710.55-156684.74) | 0.43%  (0.42 to 0.45) | 2239517.17(2225680.57-2253888.33) | 2621557.09(2605064.43-2644144.86) | 0.17%  (0.16 to 0.18) |
| Brazil | 539161.89(534359.46-544350.14) | 1069948.08(1045276.39-1094234.24) | 0.98%  (0.94 to 1.03) | 15766114.75(15448646.13-16066962.21) | 25346410.95(24761158.52-25916945.08) | 0.61%  (0.56 to 0.65) |
| Canada | 167859.36(166861.95-169080.99) | 248740.94(245417.82-252491.67) | 0.48%  (0.46 to 0.50) | 3481511.33(3461767.39-3501383.00) | 4389878.46(4327142.99-4455497.70) | 0.26%  (0.24 to 0.28) |
| China | 6289333.73(5663762.07-6939996.27) | 10642431.36(9048670.21-12216956.86) | 0.69%  (0.40 to 1.06) | 175797061.49(158321264.93-195184826.14) | 211724087.10(177375796.86-246500384.47) | 0.20%  (-0.02 to 0.48) |
| European Union | 3851593.92(3838066.60-3872005.84) | 4213527.84(4135559.76-4295221.89) | 0.09%  (0.07 to 0.11) | 77629516.41(77398461.42-77921537.81) | 69423027.92(67848821.46-70866694.04) | -0.11%  (-0.13 to -0.09) |
| France | 440096.03(436947.13-444820.44) | 508570.83(501607.58-519072.75) | 0.16%  (0.14 to 0.17) | 8396067.07(8346597.14-8467755.33) | 8076423.48(7969762.53-8203041.29) | -0.04%  (-0.05 to -0.02) |
| Germany | 842239.22(838567.93-846524.39) | 859310.77(852176.97-869879.33) | 0.02%  (0.01 to 0.03) | 16111438.38(16043860.59-16184358.60) | 13782025.01(13666518.26-13926285.36) | -0.14%  (-0.15 to -0.14) |
| India | 2879924.00(2556061.26-3114754.51) | 6471469.12(5979897.15-7033436.52) | 1.25%  (1.00 to 1.53) | 98078361.57(88626425.18-105199476.82) | 167075327.55(153991506.14-182017127.52) | 0.70%  (0.53 to 0.92) |
| Indonesia | 626372.86(548903.83-685754.94) | 1433564.78(1197605.44-1635562.55) | 1.29%  (0.90 to 1.67) | 21310359.11(18781156.31-23168456.59) | 39170615.79(33245942.46-45505819.78) | 0.84%  (0.53 to 1.19) |
| Italy | 495471.52(493178.31-498647.47) | 594285.61(588575.57-601561.95) | 0.20%  (0.19 to 0.21) | 9573470.30(9533731.88-9620009.82) | 8736869.93(8654918.07-8823013.00) | -0.09%  (-0.10 to -0.08) |
| Japan | 683550.17(678357.02-691560.68) | 1251314.02(1233194.84-1283173.02) | 0.83%  (0.81 to 0.85) | 14051184.23(13964740.67-14160082.81) | 17780905.95(17570584.34-18101855.90) | 0.27%  (0.25 to 0.28) |
| Mexico | 252295.53(249886.56-254890.01) | 622856.30(561909.61-688233.80) | 1.47%  (1.23 to 1.73) | 7926830.31(7734802.75-8143757.96) | 15509456.70(13942227.60-17208957.03) | 0.96%  (0.75 to 1.17) |
| Republic of Korea | 184903.85(176823.01-187543.79) | 267236.91(260112.10-276511.75) | 0.45%  (0.41 to 0.56) | 5086438.62(4765790.99-5199784.48) | 4768335.41(4641567.17-4959642.42) | -0.06%  (-0.09 to 0.04) |
| Russian Federation | 1396544.93(1392895.03-1399989.60) | 1571869.23(1473466.75-1668192.87) | 0.13%  (0.05 to 0.19) | 32846410.73(32752203.33-32935723.33) | 33521519.78(31227782.62-35797276.07) | 0.02%  (-0.05 to 0.09) |
| Saudi Arabia | 46632.07(37636.05-55314.93) | 103363.36(85537.48-123779.52) | 1.22%  (0.74 to 1.90) | 1705904.13(1378389.71-2062238.16) | 3370909.94(2718010.82-4134322.66) | 0.98%  (0.52 to 1.68) |
| South Africa | 112539.70(106281.56-118070.96) | 265736.27(253334.99-277713.58) | 1.36%  (1.22 to 1.51) | 3490973.00(3322660.61-3670281.72) | 6931709.12(6569775.74-7319985.39) | 0.99%  (0.85 to 1.12) |
| Turkey | 275969.42(251940.38-300237.53) | 459150.58(389187.14-533106.19) | 0.66%  (0.38 to 0.97) | 9194484.10(7903959.58-10291140.81) | 9671033.14(8122318.69-11320402.90) | 0.05%  (-0.15 to 0.30) |
| United Kingdom | 583991.76(580962.80-588283.61) | 520495.57(512755.17-528823.83) | -0.11% (-0.12 to -0.10) | 11263566.20(11210744.10-11319213.92) | 8858690.38(8731533.39-8978325.87) | -0.21%  (-0.22 to -0.20) |
| United States of America | 1861395.53(1853988.68-1872806.49) | 2633899.91(2603430.96-2667645.48) | 0.42%  (0.40 to 0.43) | 38656575.67(38533287.85-38820518.65) | 51268041.46(50695075.16-51846605.59) | 0.33%  (0.31 to 0.34) |

**Supplementary Table 3 Age-standardized mortality rate and YLL rate from Level 3 causes of NCDs in G20 countries, 1990-2021**

|  | **Mortality rate per 100000 population** | | | **YLL rate per 100000 population** | | |
| --- | --- | --- | --- | --- | --- | --- |
|  | 1990 | 2021 | Percentage change, 1990-2021 | 1990 | 2021 | Percentage change, 1990-2021 |
| Neoplasms | 157.84 (148.44-164.85) | 120.39 (109.51-129.94) | -23.73%  (-29.60 to -17.09) | 4105.39  (3897.08-4294.84) | 2901.33 (2688.91-3138.27) | -29.33%  (-34.92 to -22.50) |
| Lip and oral cavity cancer | 2.35 (2.23-2.47) | 2.31 (2.10-2.50) | -4.15% (-16.79 to 5.61) | 65.36 (62.14-68.77) | 62.65 (56.64-68.19) | -4.15% (-16.79 to 5.61) |
| Nasopharynx cancer | 1.64 (1.45-1.82) | 0.85 (0.75-0.98) | -51.05% (-58.47 to -41.24) | 56.11 (49.92-62.20) | 27.46 (24.14-31.60) | -51.05% (-58.47 to -41.24) |
| Other pharynx cancer | 1.12 (1.04-1.22) | 1.19 (1.09-1.28) | 0.88% (-10.02 to 12.71) | 33.39 (30.98-36.35) | 33.69 (30.93-36.54) | 0.88% (-10.02 to 12.71) |
| Esophageal cancer | 10.07 (8.97-11.15) | 6.85 (5.93-7.82) | -39% (-49.92 to -26.65) | 261.63 (231.32-291.74) | 159.59 (138.18-184.73) | -39% (-49.92 to -26.65) |
| Stomach cancer | 24.01 (21.87-26.64) | 11.94 (10.20-13.77) | -54.57% (-60.65 to -46.6) | 606.91 (543.07-672.15) | 275.75 (236.27-323.24) | -54.57% (-60.65 to -46.6) |
| Colon and rectum cancer | 16.96 (15.70-17.81) | 12.99 (11.65-14.06) | -24.39% (-31.81 to -16.38) | 374.82 (351.34-394.99) | 283.42 (257.99-307.93) | -24.39% (-31.81 to -16.38) |
| Liver cancer | 5.75 (5.26-6.28) | 5.59 (5.00-6.32) | -14.82% (-28.35 to 0.65) | 169.89 (153.58-187.17) | 144.71 (128.00-165.84) | -14.82% (-28.35 to 0.65) |
| Gallbladder and biliary tract cancer | 2.88 (2.59-3.16) | 2.11 (1.77-2.39) | -29.01% (-38.83 to -21.5) | 61.40 (55.14-67.98) | 43.59 (35.93-49.95) | -29.01% (-38.83 to -21.5) |
| Pancreatic cancer | 6.41 (6.02-6.72) | 6.58 (5.94-7.12) | -1.53% (-11.23 to 8.1) | 145.15 (137.98-152.49) | 142.93 (131.13-154.29) | -1.53% (-11.23 to 8.1) |
| Larynx cancer | 2.11 (1.97-2.25) | 1.29 (1.19-1.40) | -42.39% (-48.19 to -36.18) | 56.98 (53.18-60.89) | 32.83 (30.24-35.82) | -42.39% (-48.19 to -36.18) |
| Tracheal, bronchus, and lung cancer | 30.48 (28.74-32.00) | 26.23 (23.37-29.11) | -22.13% (-31.95 to -11.42) | 755.12 (712.56-795.75) | 588.00 (522.07-658.25) | -22.13% (-31.95 to -11.42) |
| Malignant skin melanoma | 0.93 (0.87-0.96) | 0.78 (0.71-0.83) | -24.45% (-30.47 to -19.95) | 25.24 (23.65-26.28) | 19.07 (17.18-20.37) | -24.45% (-30.47 to -19.95) |
| Non-melanoma skin cancer | 0.71 (0.64-0.78) | 0.72 (0.62-0.81) | -3.61% (-13.6 to 6.14) | 14.39 (13.28-15.87) | 13.87 (12.32-15.59) | -3.61% (-13.6 to 6.14) |
| Soft tissue and other extraosseous sarcomas | 0.69 (0.57-0.75) | 0.56 (0.49-0.64) | -27.64% (-33.63 to -17.55) | 23.47 (19.01-26.09) | 16.98 (14.62-19.41) | -27.64% (-33.63 to -17.55) |
| Malignant neoplasm of bone and articular cartilage | 0.75 (0.70-0.88) | 0.73 (0.57-0.84) | -4.21% (-24.65 to 13.38) | 28.17 (25.95-33.52) | 26.98 (21.31-30.68) | -4.21% (-24.65 to 13.38) |
| Breast cancer | 9.43 (8.76-9.91) | 7.43 (6.78-8.06) | -19.34% (-25.03 to -12.58) | 252.05 (237.80-266.82) | 203.31 (187.47-220.33) | -19.34% (-25.03 to -12.58) |
| Cervical cancer | 4.46 (4.09-4.84) | 2.90 (2.63-3.18) | -34.99% (-42.11 to -26.59) | 143.10 (130.31-155.76) | 93.03 (84.33-101.84) | -34.99% (-42.11 to -26.59) |
| Uterine cancer | 1.49 (1.32-1.61) | 1.13 (0.99-1.24) | -26.37% (-33.61 to -17.56) | 35.32 (30.54-38.76) | 26.00 (23.20-28.94) | -26.37% (-33.61 to -17.56) |
| Ovarian cancer | 2.84 (2.64-3.03) | 2.16 (1.95-2.35) | -22.66% (-32.86 to -14.38) | 73.37 (67.78-79.36) | 56.75 (52.26-61.88) | -22.66% (-32.86 to -14.38) |
| Prostate cancer | 6.23 (5.82-6.51) | 4.75 (4.34-5.13) | -25.36% (-29.25 to -20.4) | 101.83 (95.60-106.40) | 76.01 (69.77-82.59) | -25.36% (-29.25 to -20.4) |
| Testicular cancer | 0.17 (0.16-0.18) | 0.14 (0.13-0.15) | -15.79% (-22.82 to -8.43) | 7.59 (7.17-8.02) | 6.39 (6.02-6.78) | -15.79% (-22.82 to -8.43) |
| Kidney cancer | 2.19 (2.10-2.26) | 2.03 (1.87-2.15) | -16.59% (-21.49 to -11.64) | 55.92 (53.96-57.68) | 46.64 (43.90-49.18) | -16.59% (-21.49 to -11.64) |
| Bladder cancer | 3.72 (3.43-3.92) | 2.75 (2.47-2.99) | -31.67% (-37.98 to -23.1) | 70.96 (64.94-74.97) | 48.48 (44.35-52.82) | -31.67% (-37.98 to -23.1) |
| Brain and central nervous system cancer | 3.37 (2.89-3.78) | 3.26 (2.85-3.70) | -15.56% (-26.22 to -2.6) | 133.09 (111.05-150.18) | 112.38 (97.52-130.80) | -15.56% (-26.22 to -2.6) |
| Eye cancer | 0.12 (0.10-0.13) | 0.08 (0.06-0.09) | -40.39% (-51.52 to -28.13) | 4.94 (3.51-6.09) | 2.95 (2.17-3.61) | -40.39% (-51.52 to -28.13) |
| Neuroblastoma and other peripheral nervous cell tumors | 0.06 (0.05-0.06) | 0.07 (0.06-0.08) | 11.37% (-5.85 to 31.76) | 3.41 (2.96-3.83) | 3.80 (3.18-4.31) | 11.37% (-5.85 to 31.76) |
| Thyroid cancer | 0.56 (0.52-0.61) | 0.49 (0.43-0.53) | -13.12% (-23 to -4.56) | 13.68 (12.83-15.07) | 11.88 (10.50-13.04) | -13.12% (-23 to -4.56) |
| Mesothelioma | 0.45 (0.41-0.48) | 0.39 (0.36-0.41) | -17.1% (-26.23 to -9.32) | 10.34 (9.64-11.25) | 8.57 (8.06-9.12) | -17.1% (-26.23 to -9.32) |
| Hodgkin lymphoma | 0.59 (0.46-0.66) | 0.25 (0.21-0.32) | -58.44% (-63.66 to -47.54) | 23.03 (17.37-26.06) | 9.57 (7.77-12.52) | -58.44% (-63.66 to -47.54) |
| Non-Hodgkin lymphoma | 3.71 (3.48-4.01) | 3.05 (2.78-3.28) | -27.13% (-33.25 to -20.65) | 107.01 (100.26-116.46) | 77.98 (71.90-84.56) | -27.13% (-33.25 to -20.65) |
| Multiple myeloma | 1.44 (1.35-1.53) | 1.48 (1.31-1.60) | 1.16% (-9.28 to 10.41) | 30.46 (28.68-32.58) | 30.81 (27.40-33.75) | 1.16% (-9.28 to 10.41) |
| Leukemia | 5.93 (5.33-6.43) | 3.91 (3.44-4.29) | -45.79% (-52.2 to -36.67) | 239.54 (204.67-272.02) | 129.86 (109.56-144.46) | -45.79% (-52.2 to -36.67) |
| Other malignant neoplasms | 3.55 (3.16-3.86) | 2.62 (2.35-2.85) | -34.43% (-41.21 to -24.24) | 109.76 (93.20-120.76) | 71.97 (64.10-78.79) | -34.43% (-41.21 to -24.24) |
| Other neoplasms | 0.68 (0.58-0.84) | 0.79 (0.67-0.90) | 12.28% (-9.35 to 31.93) | 11.95 (10.17-15.53) | 13.42 (11.65-16.25) | 12.28% (-9.35 to 31.93) |
| Cardiovascular diseases | 344.11 (318.51-359.02) | 218.13 (195.80-235.27) | -36.61%  (-41.06 to -31.76) | 6786.04  (6429.08-7073.83) | 4262.88 (3960.77-4567.56) | -37.18%  (-41.45 to -31.87) |
| Rheumatic heart disease | 10.89 (9.25-12.69) | 4.62 (3.95-5.45) | -57.58% (-66.24 to -48.14) | 333.72 (280.84-391.30) | 129.71 (111.02-152.23) | -61.13% (-68.86 to -52.76) |
| Ischemic heart disease | 151.45 (140.25-157.79) | 99.96 (90.44-106.96) | -34.00% (-37.58 to -29.96) | 2890.58 (2750.01-3001.16) | 1985.97 (1836.50-2118.16) | -31.30% (-35.54 to -26.65) |
| Stroke | 139.23 (128.75-147.75) | 81.77 (72.54-89.53) | -41.27% (-46.53 to -35.11) | 2744.30 (2554.74-2918.20) | 1561.29 (1420.47-1710.22) | -43.11% (-48.70 to -36.54) |
| Hypertensive heart disease | 18.41 (15.00-20.41) | 14.06 (11.83-15.86) | -23.63% (-33.94 to -5.18) | 341.62 (274.02-380.17) | 237.42 (201.48-267.91) | -30.50% (-40.16 to -11.25) |
| Non-rheumatic valvular heart disease | 2.98 (2.69-3.17) | 2.49 (2.08-2.72) | -16.46% (-23.03 to -12.26) | 48.02 (44.58-51.22) | 35.65 (31.52-38.15) | -25.76% (-30.70 to -21.92) |
| Cardiomyopathy and myocarditis | 7.90 (7.10-8.43) | 4.62 (4.24-4.92) | -41.47% (-45.92 to -36.74) | 182.21 (165.76-196.87) | 125.66 (117.96-133.90) | -31.03% (-37.83 to -23.85) |
| Pulmonary Arterial Hypertension | 0.36 (0.30-0.42) | 0.28 (0.23-0.32) | -21.91% (-36.02 to -6.86) | 12.49 (10.27-14.21) | 7.49 (6.24-8.63) | -40.03% (-50.89 to -26.64) |
| Atrial fibrillation and flutter | 4.32 (3.74-4.73) | 4.37 (3.65-4.80) | 1.31% (-7.40 to 10.04) | 54.82 (48.59-60.13) | 54.68 (47.22-59.58) | -0.25% (-9.26 to 9.17) |
| Aortic aneurysm | 2.76 (2.56-2.90) | 1.91 (1.70-2.06) | -30.96% (-34.77 to -27.78) | 52.52 (49.71-55.30) | 37.24 (34.48-39.86) | -29.10% (-33.65 to -25.39) |
| Lower extremity peripheral arterial disease | 1.45 (1.31-1.56) | 0.87 (0.76-0.94) | -40.03% (-42.96 to -37.27) | 21.22 (19.51-22.59) | 12.98 (11.73-13.94) | -38.84% (-41.87 to -36.14) |
| Endocarditis | 0.91 (0.82-0.98) | 0.90 (0.80-0.99) | -0.70% (-8.72 to 5.19) | 24.60 (20.77-27.31) | 21.36 (19.61-24.10) | -13.15% (-22.86 to -0.77) |
| Other cardiovascular and circulatory diseases | 3.46 (3.17-3.74) | 2.28 (2.06-2.46) | -34.14% (-39.89 to -29.70) | 79.94 (72.29-86.47) | 53.44 (49.48-57.50) | -33.15% (-38.54 to -24.61) |
| Chronic respiratory diseases | 89.28 (80.87-95.97) | 55.98 (50.04-61.89) | -37.30%  (-44.13 to -27.32) | 1737.26  (1568.30-1870.59) | 996.93  (911.07-1098.62) | -42.61%  (-48.80 to -32.48) |
| Chronic obstructive pulmonary disease | 78.29 (69.98-84.68) | 48.26 (42.91-53.46) | -38.37% (-45.19 to -28.64) | 1442.26 (1288.40-1564.25) | 813.75 (733.94-899.93) | -43.58% (-50.13 to -34.12) |
| Pneumoconiosis | 0.52 (0.47-0.58) | 0.24 (0.21-0.28) | -53.94% (-61.85 to -44.45) | 11.87 (10.50-13.43) | 4.95 (4.18-5.90) | -58.33% (-66.99 to -48.19) |
| Asthma | 8.08 (6.45-11.02) | 4.47 (3.55-5.98) | -44.72% (-52.81 to -32.99) | 212.80 (173.55-275.23) | 110.79 (89.94-149.72) | -47.94% (-54.76 to -35.37) |
| Interstitial lung disease and pulmonary sarcoidosis | 1.56 (1.30-1.93) | 2.47 (2.12-2.78) | 58.32% (39.26 to 82.01) | 33.42 (26.90-42.80) | 45.85 (39.54-52.61) | 37.21% (18.87 to 62.40) |
| Other chronic respiratory diseases | 0.83 (0.75-0.95) | 0.55 (0.45-0.69) | -33.06% (-44.06 to -13.88) | 36.91 (32.11-42.56) | 21.60 (17.74-27.53) | -41.47% (-52.30 to -18.05) |
| Digestive diseases | 42.63 (39.10-47.17) | 26.36 (23.12-29.43) | -38.17%  (-49.05 to -28.02) | 1264.13  (1171.97-1397.51) | 741.13  (655.58-833.06) | -41.37%  (-51.83 to -31.81) |
| Cirrhosis and other chronic liver diseases | 20.96 (19.18-23.40) | 14.12 (12.36-16.14) | -32.63% (-45.62 to -20.88) | 694.06 (637.72-772.58) | 458.93 (403.99-525.53) | -33.88% (-46.42 to -22.74) |
| Upper digestive system diseases | 7.66 (6.89-8.51) | 2.75 (2.24-3.31) | -64.07% (-72.55 to -56.11) | 197.50 (176.28-219.91) | 62.70 (50.51-76.52) | -68.25% (-76.02 to -60.52) |
| Appendicitis | 0.78 (0.64-0.92) | 0.32 (0.26-0.39) | -58.79% (-70.28 to -46.05) | 32.31 (26.08-38.00) | 12.15 (10.00-14.99) | -62.39% (-72.54 to -50.30) |
| Paralytic ileus and intestinal obstruction | 3.79 (3.30-4.27) | 2.56 (2.18-2.92) | -32.30% (-46.06 to -20.32) | 119.80 (99.95-136.69) | 68.00 (56.81-78.36) | -43.23% (-55.68 to -32.98) |
| Inguinal, femoral, and abdominal hernia | 1.04 (0.91-1.21) | 0.55 (0.47-0.65) | -47.31% (-58.05 to -36.96) | 28.50 (24.13-34.77) | 12.48 (11.02-15.33) | -56.22% (-62.64 to -44.77) |
| Inflammatory bowel disease | 0.66 (0.57-0.72) | 0.56 (0.48-0.62) | -14.98% (-24.62 to -2.74) | 15.19 (13.09-16.94) | 11.04 (9.98-12.22) | -27.29% (-35.50 to -16.67) |
| Vascular intestinal disorders | 1.86 (1.69-2.00) | 1.15 (1.01-1.24) | -38.39% (-43.13 to -34.08) | 31.63 (29.44-34.51) | 19.59 (17.93-21.02) | -38.06% (-43.61 to -33.41) |
| Gallbladder and biliary diseases | 2.24 (1.81-2.52) | 1.52 (1.29-1.84) | -32.08% (-42.83 to -9.72) | 43.16 (32.96-49.05) | 26.18 (23.16-31.68) | -39.33% (-49.98 to -11.99) |
| Pancreatitis | 1.62 (1.45-1.84) | 1.40 (1.24-1.60) | -13.74% (-25.95 to 0.70) | 48.97 (43.74-57.51) | 42.05 (37.76-48.64) | -14.14% (-25.47 to 0.89) |
| Other digestive diseases | 2.03 (1.80-2.37) | 1.43 (1.22-1.63) | -29.59% (-43.07 to -15.10) | 53.01 (46.26-60.88) | 27.99 (24.59-32.29) | -47.20% (-56.56 to -33.21) |
| Neurological disorders | 33.89 (14.65-75.86) | 33.92 (15.20-73.19) | 0.10%  (-5.11 to 6.94) | 533.16  (297.94-1029.38) | 504.45  (276.21-996.53) | -5.39%  (-11.59 to 0.28) |
| Alzheimer's disease and other dementias | 26.06 (6.58-68.50) | 26.01 (6.95-65.88) | -0.18% (-5.21 to 7.36) | 318.33 (77.73-821.98) | 318.00 (81.66-813.51) | -0.11% (-5.55 to 7.76) |
| Parkinson's disease | 4.63 (4.20-4.92) | 4.81 (4.25-5.21) | 3.93% (-3.62 to 11.01) | 69.37 (63.65-73.80) | 69.85 (62.89-75.89) | 0.69% (-6.91 to 8.22) |
| Idiopathic epilepsy | 1.84 (1.48-2.01) | 1.38 (1.14-1.51) | -25.02% (-31.37 to -18.53) | 101.17 (78.76-111.67) | 65.75 (52.74-73.16) | -35.01% (-41.07 to -27.42) |
| Multiple sclerosis | 0.24 (0.24-0.25) | 0.21 (0.20-0.22) | -12.89% (-17.68 to -8.57) | 7.88 (7.65-8.10) | 6.12 (5.83-6.34) | -22.31% (-26.01 to -18.63) |
| Motor neuron disease | 0.48 (0.45-0.50) | 0.56 (0.52-0.61) | 18.24% (9.53 to 28.34) | 13.70 (12.53-14.69) | 14.79 (13.55-16.08) | 8.00% (-2.11 to 19.39) |
| Other neurological disorders | 0.64 (0.61-0.67) | 0.95 (0.85-1.01) | 47.62% (33.89 to 58.04) | 22.71 (21.80-23.97) | 29.94 (26.66-32.16) | 31.84% (14.21 to 44.94) |
| Mental disorders | 0.00 (0.00-0.00) | 0.00 (0.00-0.00) | 13.65%  (4.28 to 23.95) | 0.21 (0.18-0.23) | 0.24 (0.20-0.27) | 13.52%  (4.29 to 23.96) |
| Eating disorders | 0.00 (0.00-0.00) | 0.00 (0.00-0.00) | 13.65% (4.28 to 23.95) | 0.21 (0.18-0.23) | 0.24 (0.20-0.27) | 13.52% (4.29 to 23.96) |
| Substance use disorders | 3.86 (3.66-4.05) | 4.09 (3.83-4.33) | 5.87%  (-1.64 to 13.30) | 166.51  (157.49-175.45) | 177.31  (166.34-187.30) | 6.49%  (-1.23 to 14.17) |
| Alcohol use disorders | 2.39 (2.26-2.48) | 1.95 (1.58-2.14) | -18.24% (-28.98 to -9.54) | 95.78 (90.66-99.33) | 75.47 (59.80-83.33) | -21.21% (-32.51 to -12.11) |
| Drug use disorders | 1.47 (1.36-1.60) | 2.13 (2.00-2.29) | 45.09% (30.07 to 64.58) | 70.72 (65.27-77.17) | 101.85 (95.62-108.92) | 44.00% (29.13 to 63.14) |
| Diabetes and kidney diseases | 28.83 (26.95-30.38) | 31.80 (29.09-33.81) | 10.28%  (2.37 to 17.20) | 675.71  (637.95-712.85) | 692.93  (645.85-735.78) | 2.55%  (-5.06 to 9.50) |
| Diabetes mellitus | 15.81 (14.69-16.58) | 16.17 (14.91-17.20) | 2.28% (-5.10 to 9.39) | 351.52 (331.54-367.82) | 354.38 (330.13-375.64) | 0.81% (-7.02 to 8.42) |
| Chronic kidney disease | 12.69 (11.71-13.87) | 15.49 (13.85-16.84) | 22.04% (9.17 to 30.97) | 311.37 (289.56-338.41) | 334.65 (307.28-361.21) | 7.48% (-5.11 to 17.21) |
| Acute glomerulonephritis | 0.33 (0.26-0.42) | 0.14 (0.10-0.18) | -57.70% (-67.72 to -47.39) | 12.82 (10.24-15.77) | 3.90 (2.76-4.97) | -69.56% (-76.99 to -62.39) |
| Skin and subcutaneous diseases | 1.33 (1.20-1.44) | 1.33 (1.20-1.43) | -0.46%  (-9.90 to 11.82) | 39.98 (36.19-43.51) | 32.66  (30.12-35.32) | -18.33%  (-26.45 to -7.19) |
| Bacterial skin diseases | 0.76 (0.62-0.84) | 0.90 (0.81-1.01) | 18.25% (4.25 to 40.30) | 27.88 (22.49-31.21) | 23.58 (19.97-26.37) | -15.41% (-25.57 to -0.50) |
| Decubitus ulcer | 0.47 (0.41-0.52) | 0.35 (0.27-0.39) | -25.32% (-34.64 to -16.73) | 7.75 (6.47-9.06) | 6.32 (4.83-7.34) | -18.41% (-28.93 to -7.32) |
| Other skin and subcutaneous diseases | 0.11 (0.09-0.18) | 0.08 (0.07-0.15) | -24.66% (-37.47 to -7.31) | 4.36 (3.18-7.68) | 2.75 (2.09-5.27) | -36.79% (-52.12 to -12.21) |
| Musculoskeletal disorders | 1.66 (1.51-1.77) | 1.48 (1.29-1.63) | -11.04%  (-19.69 to -3.66) | 42.23 (38.65-45.30) | 34.29  (30.50-37.63) | -18.81%  (-27.52 to -11.30) |
| Rheumatoid arthritis | 0.68 (0.60-0.74) | 0.49 (0.40-0.55) | -27.94% (-35.72 to -20.97) | 14.00 (12.54-15.53) | 9.31 (7.79-10.53) | -33.52% (-40.78 to -26.83) |
| Other musculoskeletal disorders | 0.98 (0.90-1.06) | 0.99 (0.86-1.09) | 0.62% (-9.75 to 9.30) | 28.23 (25.84-30.54) | 24.98 (21.94-27.53) | -11.51% (-22.02 to -3.02) |
| Other non-communicable diseases | 20.30 (16.51-23.61) | 12.72 (11.56-14.21) | -37.33%  (-47.03 to -15.45) | 1413.71  (1063.65-1715.78) | 673.86 (584.95-793.17) | -52.33%  (-61.78 to -26.38) |
| Congenital birth defects | 13.03 (9.13-16.53) | 5.57 (4.63-6.90) | -57.26% (-66.91 to -26.00) | 1130.51 (784.70-1439.37) | 473.84 (390.82-592.31) | -58.09% (-67.85 to -26.22) |
| Urinary diseases and male infertility | 3.32 (3.00-3.58) | 4.06 (3.62-4.38) | 22.18% (10.55 to 29.68) | 80.12 (70.22-86.93) | 86.02 (76.00-93.66) | 7.36% (-3.00 to 15.38) |
| Gynecological diseases | 0.09 (0.07-0.12) | 0.09 (0.07-0.10) | -1.74% (-29.24 to 25.04) | 3.04 (2.42-4.19) | 2.99 (2.14-3.48) | -1.46% (-31.79 to 29.36) |
| Hemoglobinopathies and hemolytic anemias | 1.03 (0.91-1.12) | 0.50 (0.44-0.55) | -51.46% (-55.33 to -46.55) | 47.18 (39.92-54.12) | 19.42 (16.20-21.53) | -58.84% (-64.20 to -50.18) |
| Endocrine, metabolic, blood, and immune disorders | 1.87 (1.70-2.04) | 2.20 (1.97-2.37) | 17.76% (5.13 to 27.83) | 67.58 (58.15-76.82) | 64.83 (56.32-70.12) | -4.07% (-20.57 to 6.88) |
| Sudden infant death syndrome | 0.95 (0.65-1.36) | 0.30 (0.19-0.42) | -68.62% (-79.72 to -54.45) | 85.29 (58.15-121.72) | 26.76 (17.34-37.55) | -68.62% (-79.72 to -54.45) |

| Supplementary Table 4 Sociodemographic Index (SDI) Levels of G20 Countries in 1990 and 2021 | | |
| --- | --- | --- |
| **Countries** | **SDI (95% uncertainty intervals [UIs])** | |
|  | **1990** | **2021** |
| Argentina | 0.587 | 0.723 |
| Australia | 0.726 | 0.844 |
| Brazil | 0.500 | 0.653 |
| Canada | 0.782 | 0.873 |
| China | 0.459 | 0.722 |
| France | 0.731 | 0.838 |
| Germany | 0.817 | 0.903 |
| India | 0.333 | 0.575 |
| Indonesia | 0.457 | 0.657 |
| Italy | 0.706 | 0.806 |
| Japan | 0.790 | 0.871 |
| Mexico | 0.505 | 0.665 |
| Republic of Korea | 0.692 | 0.887 |
| Russian Federation | 0.672 | 0.809 |
| Saudi Arabia | 0.539 | 0.815 |
| South Africa | 0.542 | 0.680 |
| Turkey | 0.462 | 0.713 |
| United Kingdom | 0.744 | 0.859 |
| United States of America | 0.764 | 0.862 |

**Supplementary Table 5** Absolute and relative cross-country inequality in ASMRs and YLL rates for NCDs among G20 countries.

| Sex | years | Indexes | Age-standardized mortality rate | P Value | Age-standardized YLL rate | *P* value |
| --- | --- | --- | --- | --- | --- | --- |
| Male | 1990 | Concentration index | 0.01(-0.06 to 0.07) | 0.85 | -0.02(-0.07 to 0.03) | 0.45 |
|  | 2021 | Concentration index | -0.04(-0.09 to 0.01) | 0.10 | -0.07(-0.11 to -0.03) | **0.01** |
|  | 1990 | Slope index of inequality | -44.44(-386.08 to 297.20) | **0.59** | -6006.38(-14716.27 to 2703.50) | 0.90 |
|  | 2021 | Slope index of inequality | -402.32(-663.53 to -141.11) | 0.95 | -10694.85(-15540.46 to -5849.25) | 0.54 |
| Female | 1990 | Concentration index | -0.04(-0.10 to 0.02) | 0.17 | -0.08(-0.12 to -0.03) | **0.01** |
|  | 2021 | Concentration index | -0.08(-0.13 to -0.04) | **<0.001** | -0.12(-0.16 to -0.07) | **<0.001** |
|  | 1990 | Slope index of inequality | -293.57(-540.84 to -46.29) | 0.34 | -11208.13(-16960.86 to -5455.41) | 0.43 |
|  | 2021 | Slope index of inequality | -361.94(-557.56 to -166.33) | 0.97 | -8982.88(-13543.30 to -4422.46) | 0.86 |
| Both | 1990 | Concentration index | -0.02(-0.08 to 0.04) | 0.45 | -0.05(-0.10 to 0.00) | **0.05** |
|  | 2021 | Concentration index | -0.06(-0.11 to -0.02) | **0.01** | -0.09(-0.13 to -0.05) | **<0.001** |
|  | 1990 | Slope index of inequality | -222.04(-491.26 to 47.18) | 0.35 | -8954.88(-15314.97 to -2594.80) | 0.55 |
|  | 2021 | Slope index of inequality | -379.40(-587.75 to -171.05) | 0.97 | -9727.32(-14526.03 to -4928.62) | 0.71 |

**Supplementary Table 6** Forecast of ASMR and age-standardized YLL rate for NCDs based on disease burden from 1990 to 2021 among G20 countries, grouped by sex, projected to 2035

| **Countries** | **Sex** | **Year** | **Pred ASMR (95% CI)** | **Pred age-standardized YLL rate (95% CI)** |
| --- | --- | --- | --- | --- |
| Argentina | Both | 1990 | 689.05(667.03 to 711.07) | 15814.58(15700.09 to 15929.08) |
|  |  | 2021 | 435.94(423.17 to 448.70) | 9525.53(9456.57 to 9594.50) |
|  |  | 2035 | 206.88(61.52 to 352.25) | 4366.43(1172.86 to 7560.00) |
|  | Female | 1990 | 552.87(549.39 to 556.35) | 12308.64(12292.08 to 12325.21) |
|  |  | 2021 | 362.93(360.90 to 364.95) | 7834.36(7823.95 to 7844.76) |
|  |  | 2035 | 182.83(67.76 to 297.90) | 3662.79(1224.92 to 6100.66) |
|  | Male | 1990 | 864.99(859.72 to 870.26) | 20030.70(20006.93 to 20054.48) |
|  |  | 2021 | 531.73(528.77 to 534.69) | 11597.77(11583.92 to 11611.62) |
|  |  | 2035 | 207.43(52.88 to 361.98) | 4586.77(1309.66 to 7863.88) |
| Australia | Both | 1990 | 573.90(550.39 to 597.41) | 11978.93(11848.94 to 12108.93) |
|  |  | 2021 | 313.11(301.53 to 324.68) | 6213.11(6145.38 to 6280.84) |
|  |  | 2035 | 276.19(136.84 to 415.55) | 5528.12(2333.09 to 8723.15) |
|  | Female | 1990 | 460.59(456.79 to 464.40) | 9329.56(9310.50 to 9348.62) |
|  |  | 2021 | 258.76(256.92 to 260.60) | 4977.01(4967.12 to 4986.89) |
|  |  | 2035 | 213.53(109.45 to 317.61) | 3934.18(2288.57 to 5579.80) |
|  | Male | 1990 | 730.27(724.12 to 736.42) | 15180.97(15153.40 to 15208.54) |
|  |  | 2021 | 375.95(373.40 to 378.49) | 7566.07(7553.33 to 7578.81) |
|  |  | 2035 | 330.28(178.06 to 482.50) | 6809.77(2584.38 to 11035.16) |
| Brazil | Both | 1990 | 660.49(646.88 to 674.09) | 15630.36(15564.00 to 15696.72) |
|  |  | 2021 | 440.61(434.20 to 447.01) | 10385.76(10351.81 to 10419.70) |
|  |  | 2035 | 367.04(248.36 to 485.72) | 8050.42(5485.60 to 10615.24) |
|  | Female | 1990 | 563.12(560.79 to 565.44) | 12968.25(12957.98 to 12978.51) |
|  |  | 2021 | 377.36(376.34 to 378.38) | 8772.87(8767.76 to 8777.99) |
|  |  | 2035 | 341.84(226.29 to 457.38) | 7280.40(5022.32 to 9538.48) |
|  | Male | 1990 | 774.01(770.92 to 777.09) | 18596.52(18583.10 to 18609.93) |
|  |  | 2021 | 520.60(519.21 to 521.99) | 12299.17(12292.58 to 12305.75) |
|  |  | 2035 | 385.82(276.32 to 495.32) | 8703.52(6174.85 to 11232.19) |
| Canada | Both | 1990 | 526.67(508.50 to 544.84) | 11215.58(11118.37 to 11312.79) |
|  |  | 2021 | 324.80(314.51 to 335.09) | 6801.03(6741.49 to 6860.57) |
|  |  | 2035 | 254.80(141.71 to 367.90) | 6713.51(3000.61 to 10426.40) |
|  | Female | 1990 | 416.41(413.57 to 419.25) | 8714.77(8700.43 to 8729.10) |
|  |  | 2021 | 273.63(272.06 to 275.20) | 5556.38(5547.69 to 5565.08) |
|  |  | 2035 | 208.81(132.91 to 284.71) | 5062.92(3019.10 to 7106.74) |
|  | Male | 1990 | 680.68(676.14 to 685.22) | 14323.48(14303.06 to 14343.90) |
|  |  | 2021 | 386.58(384.45 to 388.72) | 8178.96(8167.94 to 8189.99) |
|  |  | 2035 | 291.94(148.62 to 435.25) | 7941.16(3115.79 to 12766.53) |
| China | Both | 1990 | 1150.80(1148.76 to 1152.85) | 20885.04(20855.87 to 20914.21) |
|  |  | 2021 | 764.69(763.99 to 765.38) | 10922.79(10909.88 to 10935.71) |
|  |  | 2035 | 768.74(443.49 to 1093.99) | 10805.36(4521.22 to 17089.51) |
|  | Female | 1990 | 836.81(835.61 to 838.01) | 17879.83(17875.21 to 17884.45) |
|  |  | 2021 | 437.23(436.82 to 437.64) | 8057.85(8056.03 to 8059.67) |
|  |  | 2035 | 415.45(120.93 to 709.96) | 7340.05(2060.37 to 12619.73) |
|  | Male | 1990 | 967.37(960.71 to 974.02) | 24369.66(24362.64 to 24376.68) |
|  |  | 2021 | 574.44(571.79 to 577.10) | 14281.09(14278.37 to 14283.81) |
|  |  | 2035 | 595.94(248.47 to 943.42) | 13817.91(7996.37 to 19639.44) |
| France | Both | 1990 | 518.30(506.38 to 530.21) | 15258.95(15244.69 to 15273.21) |
|  |  | 2021 | 303.10(296.03 to 310.16) | 8046.82(8038.83 to 8054.82) |
|  |  | 2035 | 221.01(126.85 to 315.16) | 6435.90(3857.42 to 9014.39) |
|  | Female | 1990 | 386.05(384.36 to 387.75) | 7675.81(7667.02 to 7684.59) |
|  |  | 2021 | 235.42(234.40 to 236.45) | 4654.90(4649.07 to 4660.73) |
|  |  | 2035 | 163.69(93.87 to 233.50) | 3226.96(1682.45 to 4771.46) |
|  | Male | 1990 | 715.24(712.05 to 718.43) | 11019.32(10955.47 to 11083.18) |
|  |  | 2021 | 390.62(389.06 to 392.18) | 6215.09(6174.63 to 6255.56) |
|  |  | 2035 | 279.23(166.01 to 392.45) | 4862.50(2644.75 to 7080.24) |
| Germany | Both | 1990 | 857.81(854.92 to 860.70) | 13706.41(13647.68 to 13765.14) |
|  |  | 2021 | 470.44(468.96 to 471.92) | 7580.10(7542.00 to 7618.21) |
|  |  | 2035 | 361.38(210.25 to 512.52) | 5814.03(3098.56 to 8569.08) |
|  | Female | 1990 | 533.42(531.82 to 535.03) | 10588.28(10579.95 to 10596.61) |
|  |  | 2021 | 319.24(318.21 to 320.27) | 6000.56(5994.91 to 6006.21) |
|  |  | 2035 | 234.23(136.10 to 332.35) | 4292.43(2122.69 to 6462.16) |
|  | Male | 1990 | 654.47(643.48 to 665.47) | 18082.84(18069.97 to 18095.71) |
|  |  | 2021 | 387.60(380.78 to 394.43) | 9344.17(9336.91 to 9351.44) |
|  |  | 2035 | 300.22(169.58 to 431.86) | 7138.39(4397.94 to 9878.84) |
| India | Both | 1990 | 662.95(656.32 to 669.58) | 18946.26(18940.50 to 18952.02) |
|  |  | 2021 | 602.78(599.14 to 606.41) | 15994.64(15991.23 to 15998.06) |
|  |  | 2035 | 465.92(183.15 to 748.68) | 11822.93(6327.37 to 17318.49) |
|  | Female | 1990 | 586.93(585.71 to 588.15) | 14891.20(14886.02 to 14896.38) |
|  |  | 2021 | 520.00(519.38 to 520.62) | 11728.09(11725.32 to 11730.85) |
|  |  | 2035 | 371.89(127.11 to 616.67) | 8011.68(2726.94 to 13296.43) |
|  | Male | 1990 | 736.07(734.70 to 737.44) | 16988.03(16956.86 to 17019.20) |
|  |  | 2021 | 697.20(696.40 to 698.00) | 13799.75(13782.22 to 13817.29) |
|  |  | 2035 | 525.21(281.37 to 769.05) | 10347.54(4025.11 to 16669.97) |
| Indonesia | Both | 1990 | 683.61(670.01 to 697.21) | 17361.97(17294.78 to 17429.16) |
|  |  | 2021 | 738.16(728.47 to 747.85) | 16314.01(16268.07 to 16359.95) |
|  |  | 2035 | 646.34(477.41 to 815.27) | 13840.80(10030.49 to 17651.12) |
|  | Female | 1990 | 657.51(655.04 to 659.98) | 16499.65(16488.44 to 16510.86) |
|  |  | 2021 | 661.36(659.62 to 663.10) | 14319.39(14312.10 to 14326.69) |
|  |  | 2035 | 554.53(429.11 to 679.95) | 11399.02(8674.84 to 14123.21) |
|  | Male | 1990 | 712.25(709.40 to 715.10) | 18252.72(18240.20 to 18265.23) |
|  |  | 2021 | 826.23(823.98 to 828.48) | 18440.94(18431.87 to 18450.02) |
|  |  | 2035 | 752.37(537.46 to 967.29) | 16367.08(11655.16 to 21079.00) |
| Italy | Both | 1990 | 576.12(563.77 to 588.46) | 11804.07(11737.47 to 11870.67) |
|  |  | 2021 | 330.82(323.89 to 337.75) | 6119.88(6079.22 to 6160.55) |
|  |  | 2035 | 247.25(158.02 to 336.48) | 4725.60(1707.37 to 7743.84) |
|  | Female | 1990 | 458.99(457.12 to 460.86) | 8916.23(8906.75 to 8925.71) |
|  |  | 2021 | 272.72(271.68 to 273.77) | 4901.16(4895.17 to 4907.15) |
|  |  | 2035 | 206.05(133.94 to 278.16) | 3775.53(1961.09 to 5589.97) |
|  | Male | 1990 | 739.31(736.30 to 742.33) | 15428.90(15415.05 to 15442.74) |
|  |  | 2021 | 407.51(405.99 to 409.03) | 7572.68(7564.94 to 7580.41) |
|  |  | 2035 | 288.71(189.81 to 387.62) | 5348.37(2025.53 to 8671.22) |
| Japan | Both | 1990 | 438.76(430.82 to 446.69) | 11560.81(11552.38 to 11569.24) |
|  |  | 2021 | 262.33(258.01 to 266.65) | 6711.06(6706.12 to 6716.00) |
|  |  | 2035 | 234.66(154.94 to 314.39) | 5956.36(4036.17 to 7876.55) |
|  | Female | 1990 | 351.50(350.27 to 352.74) | 6871.78(6865.96 to 6877.60) |
|  |  | 2021 | 197.58(196.95 to 198.21) | 3832.13(3828.22 to 3836.03) |
|  |  | 2035 | 176.67(117.45 to 235.88) | 3827.78(2464.11 to 5191.46) |
|  | Male | 1990 | 560.62(558.69 to 562.56) | 8949.52(8908.98 to 8990.07) |
|  |  | 2021 | 341.88(340.97 to 342.78) | 5176.79(5150.97 to 5202.62) |
|  |  | 2035 | 297.27(203.34 to 391.20) | 4912.15(3142.19 to 6682.11) |
| Mexico | Both | 1990 | 643.82(640.06 to 647.59) | 14532.06(14438.89 to 14625.22) |
|  |  | 2021 | 588.77(586.73 to 590.80) | 12294.73(12243.18 to 12346.29) |
|  |  | 2035 | 583.23(339.10 to 827.37) | 11521.03(6035.06 to 17006.99) |
|  | Female | 1990 | 629.42(624.85 to 633.99) | 13461.64(13444.08 to 13479.20) |
|  |  | 2021 | 465.75(464.06 to 467.44) | 10410.37(10402.51 to 10418.23) |
|  |  | 2035 | 417.61(249.32 to 585.90) | 8841.54(5119.18 to 12563.89) |
|  | Male | 1990 | 628.57(608.80 to 648.34) | 15822.36(15805.67 to 15839.05) |
|  |  | 2021 | 522.78(512.71 to 532.85) | 14398.24(14388.53 to 14407.95) |
|  |  | 2035 | 505.74(282.48 to 728.99) | 13847.04(7962.38 to 19731.69) |
| Republic of Korea | Both | 1990 | 778.78(749.74 to 807.83) | 22092.20(22053.53 to 22130.87) |
|  |  | 2021 | 295.01(286.59 to 303.44) | 7184.39(7175.25 to 7193.53) |
|  |  | 2035 | 246.16(134.49 to 357.82) | 5463.71(2841.06 to 8086.35) |
|  | Female | 1990 | 628.59(623.70 to 633.48) | 12455.30(12436.06 to 12474.53) |
|  |  | 2021 | 229.78(228.51 to 231.05) | 4032.36(4025.99 to 4038.74) |
|  |  | 2035 | 188.38(112.45 to 264.32) | 3704.87(1946.28 to 5463.45) |
|  | Male | 1990 | 1019.63(1009.67 to 1029.58) | 16573.05(16440.91 to 16705.19) |
|  |  | 2021 | 382.00(379.88 to 384.13) | 5476.22(5431.36 to 5521.09) |
|  |  | 2035 | 303.37(169.59 to 437.15) | 4694.59(2236.20 to 7152.99) |
| Russian Federation | Both | 1990 | 870.55(859.03 to 882.08) | 19362.59(19305.53 to 19419.64) |
|  |  | 2021 | 669.87(661.46 to 678.27) | 15195.26(15150.60 to 15239.93) |
|  |  | 2035 | 1343.48(0.00 to 8142.20) | 28943.46(0.00 to 169044.08) |
|  | Female | 1990 | 703.13(701.46 to 704.80) | 14284.50(14276.95 to 14292.05) |
|  |  | 2021 | 539.15(537.95 to 540.36) | 11023.45(11017.44 to 11029.46) |
|  |  | 2035 | 561.70(-741.99 to 1865.39) | 11575.52(-14341.16 to 37492.20) |
|  | Male | 1990 | 1203.66(1199.74 to 1207.58) | 27490.57(27475.19 to 27505.94) |
|  |  | 2021 | 856.92(854.84 to 859.00) | 20699.34(20689.41 to 20709.26) |
|  |  | 2035 | 618.50(-966.04 to 2203.04) | 15151.40(-22720.38 to 53023.17) |
| Saudi Arabia | Both | 1990 | 823.55(768.37 to 878.73) | 18975.04(18705.91 to 19244.17) |
|  |  | 2021 | 657.04(623.61 to 690.46) | 14073.28(13921.38 to 14225.18) |
|  |  | 2035 | 622.09(328.03 to 916.15) | 15682.54(7865.24 to 23499.85) |
|  | Female | 1990 | 802.79(791.56 to 814.03) | 18498.62(18446.41 to 18550.84) |
|  |  | 2021 | 627.67(620.17 to 635.18) | 13230.32(13198.73 to 13261.91) |
|  |  | 2035 | 546.91(313.11 to 780.71) | 13448.34(7494.29 to 19402.40) |
|  | Male | 1990 | 842.11(830.54 to 853.68) | 19391.91(19342.81 to 19441.01) |
|  |  | 2021 | 675.26(668.20 to 682.31) | 14613.99(14586.13 to 14641.86) |
|  |  | 2035 | 642.12(341.56 to 942.67) | 16337.42(8488.65 to 24186.20) |
| South Africa | Both | 1990 | 557.64(532.00 to 583.28) | 13786.93(13660.60 to 13913.26) |
|  |  | 2021 | 647.79(628.70 to 666.89) | 14518.86(14427.29 to 14610.43) |
|  |  | 2035 | 801.40(72.19 to 1812.09) | 16781.02(1581.56 to 37857.40) |
|  | Female | 1990 | 488.39(484.27 to 492.52) | 12069.59(12050.61 to 12088.56) |
|  |  | 2021 | 586.26(583.10 to 589.43) | 12841.28(12827.38 to 12855.17) |
|  |  | 2035 | 662.69(-147.83 to 1473.21) | 13486.43(-3278.94 to 30251.81) |
|  | Male | 1990 | 656.84(650.84 to 662.83) | 16073.24(16047.02 to 16099.46) |
|  |  | 2021 | 735.12(730.35 to 739.88) | 16726.96(16707.36 to 16746.56) |
|  |  | 2035 | 703.39(227.34 to 1179.44) | 15208.54(4739.99 to 25677.09) |
| Turkey | Both | 1990 | 840.29(816.41 to 864.16) | 21222.73(21104.67 to 21340.78) |
|  |  | 2021 | 542.70(531.26 to 554.13) | 10982.61(10925.44 to 11039.77) |
|  |  | 2035 | 474.47(118.63 to 830.61) | 9367.78(2514.87 to 16249.38) |
|  | Female | 1990 | 671.82(667.85 to 675.80) | 16890.65(16872.66 to 16908.64) |
|  |  | 2021 | 454.60(452.65 to 456.56) | 8658.36(8649.70 to 8667.01) |
|  |  | 2035 | 365.34(56.80 to 673.88) | 6858.12(1002.27 to 12713.98) |
|  | Male | 1990 | 1036.18(1030.55 to 1041.81) | 25959.36(25934.58 to 25984.14) |
|  |  | 2021 | 644.23(641.65 to 646.82) | 13537.07(13525.62 to 13548.51) |
|  |  | 2035 | 546.92(266.68 to 827.16) | 11024.40(5445.13 to 16603.68) |
| United Kingdom | Both | 1990 | 634.84(622.10 to 647.57) | 13473.84(13405.92 to 13541.76) |
|  |  | 2021 | 366.51(358.34 to 374.67) | 7438.62(7393.56 to 7483.68) |
|  |  | 2035 | 255.83(104.74 to 407.15) | 5887.75(797.07 to 11023.05) |
|  | Female | 1990 | 517.82(515.89 to 519.75) | 10793.48(10783.40 to 10803.55) |
|  |  | 2021 | 315.16(313.89 to 316.44) | 6227.13(6220.40 to 6233.86) |
|  |  | 2035 | 207.31(104.86 to 309.77) | 4351.49(860.64 to 7842.35) |
|  | Male | 1990 | 811.30(808.15 to 814.44) | 16987.04(16972.90 to 17001.18) |
|  |  | 2021 | 426.76(425.09 to 428.43) | 8770.69(8762.33 to 8779.05) |
|  |  | 2035 | 285.78(111.87 to 459.69) | 6581.90(2011.99 to 11151.82) |
| United States of America | Both | 1990 | 574.30(567.90 to 580.70) | 12808.84(12775.37 to 12842.31) |
|  |  | 2021 | 442.64(438.15 to 447.13) | 9940.72(9916.17 to 9965.27) |
|  |  | 2035 | 435.38(228.96 to 641.80) | 10627.27(5461.67 to 15792.86) |
|  | Female | 1990 | 467.87(466.90 to 468.85) | 10201.71(10196.74 to 10206.68) |
|  |  | 2021 | 372.71(372.03 to 373.39) | 8076.56(8072.95 to 8080.17) |
|  |  | 2035 | 340.16(200.02 to 480.29) | 7904.90(4621.16 to 11188.63) |
|  | Male | 1990 | 727.38(725.86 to 728.89) | 16124.82(16117.81 to 16131.82) |
|  |  | 2021 | 526.80(525.89 to 527.71) | 12010.04(12005.41 to 12014.66) |
|  |  | 2035 | 516.08(265.95 to 766.21) | 12883.14(6600.35 to 19165.94) |
